# Consensus recommendations for clinical functional MRI applied to language mapping

**DOI:** 10.52294/001c.128149

**Published:** 2025-01-23

**Authors:** Natalie L. Voets, Manzar Ashtari, Christian F. Beckmann, Christopher F. Benjamin, Tammie Benzinger, Jeffrey R. Binder, Alberto Bizzi, Bruce Bjornson, Edward F. Chang, Linda Douw, Jodie Gawryluk, Karsten Geletneky, Matthew F. Glasser, Sven Haller, Mark Jenkinson, Jorge Jovicich, Eric Leuthardt, Asim Mian, Thomas E. Nichols, Oiwi Parker Jones, Cyril Pernet, Puneet Plaha, Monika Połczyńska-Bletsos, Cathy J. Price, Geert-Jan Rutten, Michael Scheel, Joshua S. Shimony, Joanna Sierpowska, Lynne J. Williams, Ghoufran Talib, Michael Zeineh, Andreas Bartsch, Susan Bookheimer

**Affiliations:** 1 FMRIB-WIN Centre, Nuffield Department of Clinical Neurosciences, University of Oxford; 2 Departments of Ophthalmology and Radiology, University of Pennsylvania; 3 Department of Cognitive Neuroscience & Donders Institute, Radboud University Nijmegen; 4 Vancouver General Hospital; 5 Mallinckrodt Institute of Radiology, Washington University School of Medicine; 6 Departments of Neurology and Biophysics, Medical College of Wisconsin; 7 Neuroradiology Unit, Fondazione IRCCS Istituto Neurologico Carlo Besta; 8 BC Children’s Hospital, Department of Pediatrics (Neurology), University of British Columbia; 9 Department of Neurological Surgery, University of California, San Francisco; 10 Department of Anatomy and Neurosciences, Amsterdam University Medical Centers; 11 Department of Psychology, University of Victoria, British Columbia; 12 Klinikum Darmstadt, Academic Teaching Hospital of the Universities Frankfurt/Main and Heidelberg-Mannheim; 13 Departments of Radiology and Neuroscience, Washington University School of Medicine; 14 Centre D’Imagerie Medicale de Cornavin and Faculty of Medicine, University of Geneva; 15 Australian Institute for Machine Learning (AIML), School of Computer and Mathematical Sciences, University of Adelaide; 16 South Australian Health and Medical Research Institute; 17 Center for Mind/Brain Sciences, University of Trento; 18 Department of Neurosurgery, Washington University School of Medicine; 19 School of Medicine, Boston University; 20 Big Data Institute, Li Ka Shing Centre for Health Information and Discovery, University of Oxford; 21 Nuffield Department of Clinical Neurosciences, University of Oxford; 22 Neurobiology Research Unit, Rigshospitalet; 23 Nuffield Department of Neurosurgery, University of Oxford; 24 Department of Psychiatry and Biobehavioural Sciences, University of California, Los Angeles; 25 Wellcome Centre for Human Neuroimaging, University College London; 26 Department of Neurosurgery, Elisabeth-Tweesteden Hospital Tilburg; 27 Institute of Neuroradiology, Charité - Universitätsmedizin Berlin; 28 Cognition and Brain Plasticity Unit & Neuroscience Institute, Dept. of Cognition, Development and Educational Psychology, University of Barcelona; 29 Department of Radiology, Stanford University; 30 Radiologie Bamberg; 31 Department of Neuroradiology, University of Heidelberg; 32 Department of Psychiatry and Biobehavioral Sciences, UCLA School of Medicine, University of California, Los Angeles

**Keywords:** functional MRI, language, neurosurgery, surgical planning, fMRI, brain mapping, brain / surgery, functional laterality

## Abstract

Ample reports highlight fMRI’s added value to guide neurosurgical interventions near brain regions supporting speech and language. However, fMRI’s usefulness for clinical language mapping remains controversial, partly fueled by 1) differences from clinical standard tools it is often compared against, and 2) wide heterogeneity in how data are acquired, analyzed and interpreted. Both factors limit objective assessment of the benefits and efficacy of presurgical fMRI. This underscores the need for standardization of fMRI protocols to enable data pooling across centers and facilitate learning from patient outcomes. The OHBM Working Group on clinical fMRI language mapping was formed in 2017. Its scope was to review and propose best practice recommendations addressing specific challenges posed by applications in patient populations. Objectives were to: 1) consider language tasks and designs, optimized for specific clinical objectives, and incorporating modifications for patients with existing impairments; 2) offer practical guidance, based on high-quality research, for each step from fMRI acquisition and analysis to reporting individual patients’ data. In considering these challenges we focus on implementations that have proven feasible based on approaches in active use today. When widely available practices deviate from optimal practices, we highlight emerging developments meriting further evaluation and incorporation into clinical use. This document was created in collaboration with the OHBM Committee on Best Practices, incorporating community feedback. It aims to provide a framework for improved standardization of fMRI to enable much-needed evaluations of its ultimate goals; namely, minimization of invasive intraoperative testing and, ultimately, of new post-operative language deficits. Accordingly, the single strongest recommendation is for greater transparency and reporting of longitudinal outcomes in patients undergoing clinical fMRI.

The following manuscript is a shortened summary that accompanies the full OHBM ratified ‘best practices recommendations’ available online (https://doi.org/10.31219/osf.io/r7u8p). Amendments have been made to the present manuscript to incorporate additional reviewer feedback, leading to slight divergences when compared to the officially approved Best Practice Recommendations. A quick-reference summary is presented in Box 1; supporting evidence is detailed in the following document and the extended, uncut version. All appendices referenced in this manuscript can be found in the full document.

## 1. INTRODUCTION

The main clinical applications of functional MRI (fMRI) for individual patients include preoperative *risk assessment* and the mapping of “critical” cortex^1^ for presurgical *planning*.^1^ *Risk assessment* refers to determining whether surgery will take place in the language dominant hemisphere, which entails greater risk for postoperative language impairments. Consequently, establishing how ‘strongly’ the targeted hemisphere is dominant for language informs patient consent, deciding whether to proceed with surgery, and the surgical plan itself. Presurgical *planning* also includes deciding on a strategy to reach a surgical target while minimizing damage to surrounding functionally important brain tissue. fMRI - if properly performed - allows the noninvasive visualization of gray matter functions at an individual patient level. But what constitutes ‘high-quality’ clinical fMRI?

The focus of most validation work on clinical fMRI has been mapping speech- and language-related functions, because their localization, with few exceptions (e.g., the ventral premotor cortex), cannot be accurately predicted from anatomy alone. However, despite ample reports highlighting fMRI’s value in guiding, selecting and tailoring neurosurgical interventions close to language regions, the application of fMRI for clinical language mapping remains controversial.^2,3^ While this controversy stems from several sources (including comparisons against other methods instead of patient outcome), one main challenge is the dramatic variation in approaches used in clinical language fMRI and, likely as a result, variability in outcomes.^4^

The biggest challenge for developing clinical guidelines to standardize practice is the large variety of tasks and methods^4–7^ used to conduct, analyze, and interpret clinical fMRI studies. Clinical language fMRI is more-or-less *equal parts* language neuroscience experiment, cognitive assessment, neuroradiological exam, image analysis application, statistical inference, and neurosurgical decision-making tool. The requirement for this range of complementary skills has meant that as fMRI has moved into the clinic, the discipline has fragmented among a range of professions seeking to characterize and standardize clinical fMRI from their unique perspectives,^4,8^ which may often mean a lack of interdisciplinary consensus.

In one early attempt at standardizing fMRI, neuropsychologists focused on the *skills* that valid clinical fMRI requires.^9^ A multidisciplinary approach was advocated, involving professionals with expertise in critical domains including (among others) cognitive and computer science, psychology, neuroradiology, neurology, and neurosurgery. In 2017, the American Society for Functional Neuroradiology (ASFNR) published recommendations for cognitive *tasks* used to map language functions in surgical patients. Perhaps in part due to the group’s neuroradiological focus, recommendations were based on a survey of tasks commonly in use at members’ institutions.^7^ In another report, the American Academy of Neurology summarized the *quality of evidence* supporting fMRI for lateralization^2^ of language.^5^ When representative samples of individuals collecting and analyzing fMRI for epilepsy surgical programs were surveyed in the US^4^ and the EU,^6^ essentially all aspects of the procedure were found to vary.

The fMRI *tasks* and *analysis* approaches used are the most important aspects of clinical language fMRI to standardize to ensure best patient care. It may be of interest to know what tasks are in common use, but it is more critical to consider the *validity* of various practices. Several aspects of fMRI task design (e.g., sensory modality, control conditions) are important to explicitly consider due to their influence on the specificity of the activation patterns. In patients with existing language impairments, it may furthermore be necessary to alter task conditions, speed of presentation or difficulty levels, whereas ‘standard’ tasks have traditionally been ‘one-size-fits all’. From an *analysis* perspective, there is a lack of recommendations that address state-of-the-art data collection, preprocessing, analysis, and reporting. For example, in one clinical survey,^4^ the most used fMRI data smoothing kernel was 8mm, which happens to be the default setting in a prevalent data analysis package often used in neuroscience studies.^10^ Yet, default settings in research software are generally chosen with a different outcome objective in mind, namely to maximize *group-level* sensitivity by *minimizing inter-individual variation* in fMRI activation location and extent.^11^ **In clinical settings, it is precisely the individual variability that we aim to capture because of its impact on surgical decision-making**. Such technical and methodological considerations have profound implications for the interpretability of clinical fMRI^2,3^ and the spatial ‘localizability’ of findings.^12^

Through efforts by the Organization for Human Brain Mapping (OHBM) to improve best practices for neuroscientific imaging data analysis and reproducibility, Best Practices in Data Analysis and Sharing (COBIDAS) have been recommended.^13,14^ The OHBM Clinical fMRI Working Group was formed in 2017 in response to the specific challenges posed by clinical fMRI under the COBIDAS principles. This Working Group consists of a multidisciplinary committee spanning all domains of relevant expertise, including neurologists, neuroradiologists, neurosurgeons, imaging statisticians, neuroscientists, and neuropsychologists in both adult and pediatric populations. Bringing together practitioners from North America and Europe, the working group had two main goals:

1. To present the unique challenges of mapping language functions in clinical populations who frequently suffer from neurocognitive impairments; and
2. To put forward practical guidance for the acquisition, pre-processing, analysis, and reporting of individual patient data based on high quality research.

This document summarizes the main recommendations of the Clinical fMRI Working Group. Further in-depth considerations of the presented topics are provided in the full document (https://doi.org/10.31219/osf.io/r7u8p). In light of continued progress, especially with respect to technical aspects of fMRI data collection and analysis, it is hoped that these recommendations will improve minimum standards and facilitate **meaningful use**, enhance comparability between studies, and support large-scale evaluations of fMRI’s strengths and limitations in clinical practice.

## 2. PART 1. CLINICAL INDICATIONS AND APPROACHES FOR FMRI LANGUAGE MAPPING

Due to the complexity of the language system, presurgical language fMRI starts by establishing the clinical question that fMRI is intended to answer. The following sections have been shortened; further nuances and supporting data are presented in the full document and its Appendices.

### 2.1. INCLUSION / EXCLUSION CRITERIA AND ADDITIONAL INFORMATION REQUIRED PRIOR TO FMRI

#### 2.1.1. PATIENT SELECTION

**Fig 1.**
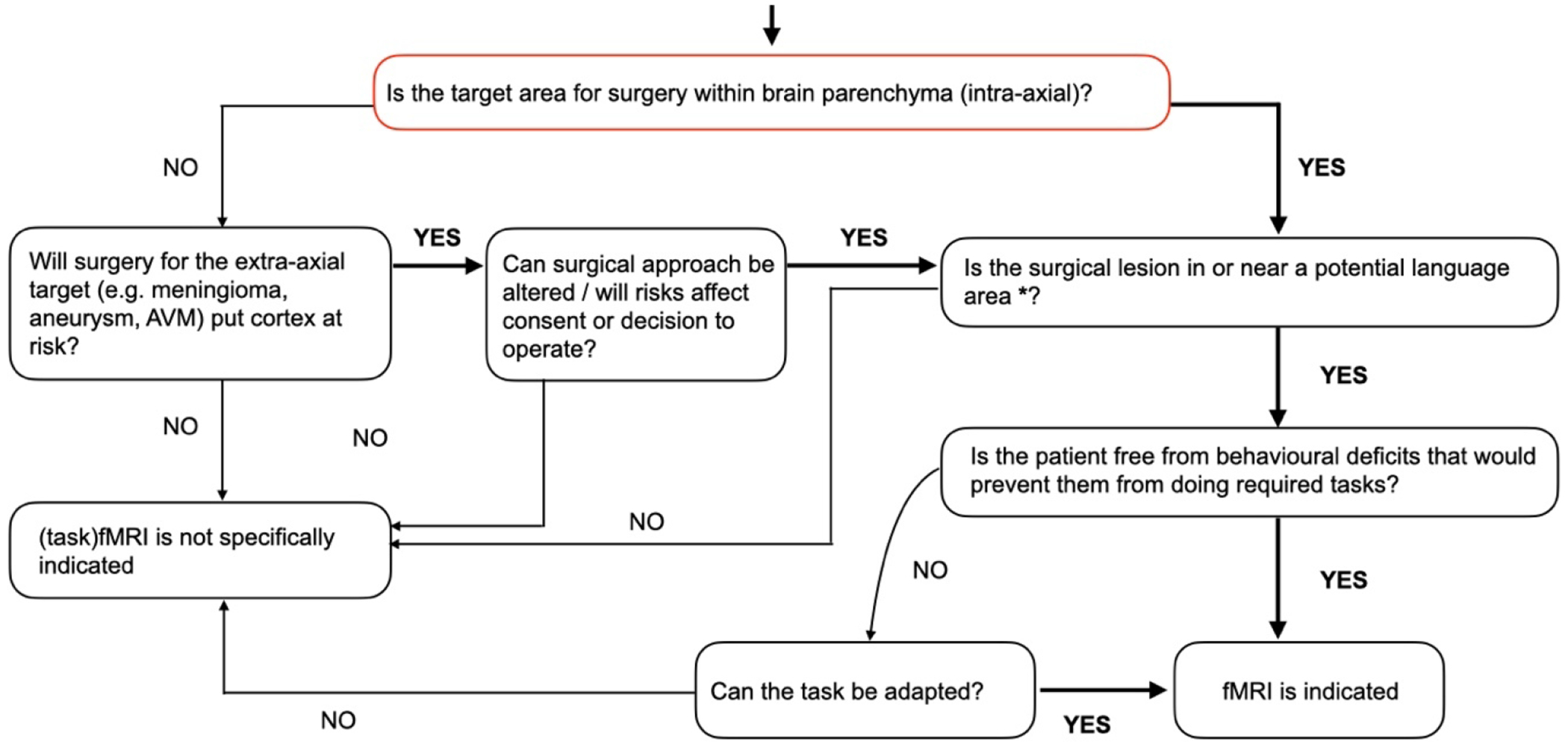
Inclusion / exclusion flowchart for presurgical language fMRI AVM = arteriovenous malformation. * See section 2.2.2 for details describing localization of language regions.

FMRI is primarily indicated for surgical targets *within* the brain parenchyma, i.e., intra-axially located, *and* where there is a risk of surgery causing language impairment. This risk may arise because the lesion is within or near functional cortex or associated white matter pathways. Alternatively, risks may exist when the surgical trajectory, e.g., a trans-opercular approach to the insula, could disrupt language networks. In selected extra-axial surgeries (e.g., resections of space occupying meningiomas, arteriovenous malformations (AVMs) with a nidus outside the brain parenchyma, or aneurysm clipping), language fMRI mapping can be helpful to inform surgical access and/or the temporal order of multi-stage embolization in relation to language territories at risk of hemorrhagic or ischemic damage^15^ (Fig 1).

#### 2.1.2. HANDEDNESS & SIDE OF SURGERY

Numerous factors influence the risk that neurosurgery poses to language functions; one is handedness. The overall likelihood of atypical (i.e., ‘bilateral’ or right-hemisphere) language dominance is higher in left-handed or ambidextrous individuals (22-30%) than in right-handed people (4-12%) (e.g.,^16–18^). Still, the majority of non-right-handed individuals have typical left-hemisphere dominance for speech and language.^19^ Consequently, handedness alone is useful to *inform*, but not sufficient to *rule out* surgical risks. The incidence of atypical (bilateral or right-hemisphere) language dominance is higher in people with chronic epilepsy^20,21^ and some with a long-standing / slow-growing left hemisphere lesion (e.g.,^22–24^). Clearly, the consequences to the patient of removing potentially language-critical structures in the right hemisphere may be severe. A case can therefore be made for pre-surgical language fMRI if a surgical target, or intended access route, approaches possible language structures ***in either hemisphere***.^25^

#### 2.1.3. COGNITIVE STATUS

Developments in resting state fMRI (rs-fMRI) offer promising opportunities for understanding the organization of functional networks without active patient participation, particularly in populations where task-based mapping is challenging^26–31^ (Appendix A of the full online report: https://doi.org/10.31219/osf.io/r7u8p). Rs-fMRI can provide valuable insights, particularly for assessing language lateralization or broad network organization. Indeed, it is widely acknowledged that functional networks extracted from rs-fMRI data typically correspond, at the group level, well with those from task-based fMRI. However, at the single-subject level, deducing the correspondence between a resting network and a specific cognitive function is less warranted, particularly in cases where – due to pathology – individual-specific organization can be expected to deviate significantly from a group-level organization of cognitive processing in the brain. For example, language laterality estimated from rs-fMRI has shown variable concordance (typically lower in patients than in controls^32^) and occasional misclassifications when compared to task-fMRI acquired in the same patients.^33–35^ Further validation of rs-fMRI is needed, as also highlighted in recent recommendations from the American Society for Neuroradiology,^36^ especially in relation to predicting individual patient language *outcomes*. **Consequently, our consensus is that task-based mapping remains the most reliable method for identifying brain regions involved in specific aspects of language processing** and that, currently, there is insufficient data to support the use of rs-fMRI alone for the purposes of mapping language processes whenever a task can be acquired.

However, task-based fMRI is often not feasible in patients who are unable to cooperate or tolerate MRI, including very young children (typically < 6 years, depending on the child) who may require sedation to undergo MRI. In select adult cases and very young children, rs-fMRI or passive tasks (e.g., sentence or story listening) may serve as useful adjuncts, particularly when adapted to the developmental and clinical context. However, there is a paucity of studies in infants assessing – with any technique – the lateralization or localization of language functions.^37^ There is similarly insufficient data reported in wider populations unable to tolerate task-based fMRI to guide specific recommendations. A small number of studies have attempted to determine language lateralization from fMRI acquired in children under deep sedation. The largest study to date concluded from the much higher rate of ‘atypical’ (bilateral) patterns that “language patterns observed in a sedated fMRI with passive task may not truly represent the language networks of wakefulness”.^38^ In children under <5 years, language networks may not be fully established and activation patterns may be particularly modulated by language abilities,^37^ adding interpretational confounds to maps without active participations and performance readouts.

Since our **recommendations were created in the context of mapping speech and language-related functions with task-based fMRI for specifically indicated purposes and populations** (Fig 1), **we are not able to put forward consensus recommendations outside of those applications and populations**, i.e., for use in individuals (child or adult) unable to actively participate in task-based fMRI. In such cases, alternative methodologies for functional localization and/ or lateralization with e.g., ESM, electrocorticography, transcranial magnetic stimulation, functional near-infrared spectroscopy and functional transcranial doppler ultrasonography may have complementary roles. Yet, as with fMRI, further data are needed around the contribution of each of these methods to predict language outcomes in these populations.^39^

For all these reasons, appreciating the detailed neurological and cognitive performance status of each patient is essential when planning language fMRI. In some patient groups, such as those with temporal lobe epilepsy, use of pre- and post-operative cognitive assessment batteries is well established and predicts whether dominant left temporal patients are at low, medium, or high risk of post-surgical naming decline.^40^ In other populations, such as high-grade gliomas, pre-operative performance is not routinely captured. However, impairments may prevent patients from completing meaningful fMRI. There is little benefit to be gained, for example, by attempting fMRI language mapping when the patient is too aphasic to comprehend instructions, or tasks are too difficult for them (see 2.3.6). As a minimum, potential **patients should (a) be co-operative and able to tolerate being in a noisy, confined space while performing fMRI tasks** and (b) have **minimum abilities to both perform required tasks and simultaneously avoid certain behaviors** (e.g., moving or talking out loud during the task, if silent responses are required). Neuropsychological assessment can (1) detect subtle deficits not apparent conversationally but predictive for surgical risks; (2) identify the need for modifications to fMRI tasks; (3) identify wider attention/memory difficulties that may affect fMRI performance. It is also important to consider deviations from age-typical milestones (whether precocious or delayed) for appropriate task selection in pediatric patients (see full document). Consequently, **best practice is to establish neuropsychological performance before fMRI and surgery, and again post-operatively** to evaluate outcomes. Short (e.g., bedside) testing of primary cognitive and language domains, along with some more targeted in-depth assessment of deficits, is generally feasible in virtually every case.

#### 2.1.4. CONTRAINDICATIONS

Absolute and relative MRI contraindications apply, while important confounds limit fMRI interpretability, considered in the full document and accompanying Appendix B.

### 2.2. CLINICAL OBJECTIVES OF PRESURGICAL FMRI LANGUAGE MAPPING

Objectives of speech and language fMRI mapping generally separate into *inter-hemispheric lateralization* versus *localization of functional tissue*. Tasks optimized to *lateralize* language can be less suited to *localize* functions, and vice versa. Consequently, precision in formulating the clinical request and surgical goals helps with tailoring tasks to maximize fMRI’s utility.

#### 2.2.1. LATERALIZING LANGUAGE FUNCTIONS

Risks of developing postoperative language and verbal memory deficits is partly dependent on language lateralization.^5,40^ Language lateralization using fMRI has been most extensively assessed prior to temporal lobe surgery to treat drug-resistant epilepsy.^21,41^ The clinical purpose is usually to establish *graded language lateralization* as an alternative to invasive Wada testing.^42^ Laterality results are often used to inform consent and/or tailor the surgical approach / extent. Laterality can also be useful to (re-)establish in patients who have undergone previous surgery, in whom inter-hemispheric language dominance may change through functional reorganization.^22^

**Fig 2.**
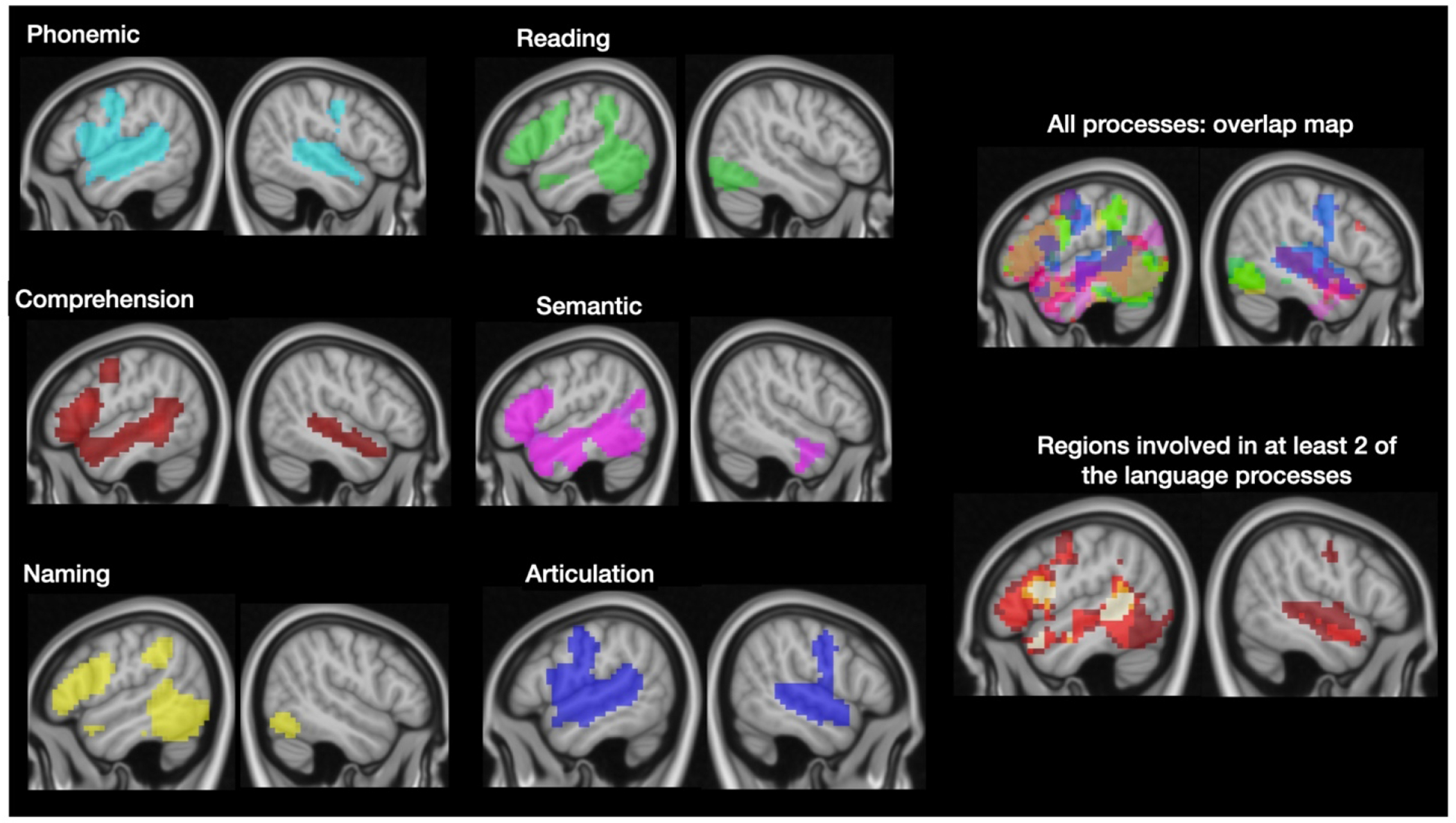
Relative lateralization of language processes based on fMRI Different language processes are lateralized to different extents, depending on the language process engaged during a given fMRI task, and – importantly – what control condition the target language process is compared against (see Fig 5). Activation results are shown from predictions based on meta-analysis of ~13,500 neuroimaging studies in Neuroquery (http://neuroquery.org), separately querying the search terms “phonemic”, “comprehension”, “naming”, “reading”, “semantic” and “articulation”. The Neuroquery-derived z-score maps are shown thresholded at z=3.1 (corresponding to p < 0.01). Overlap maps show all processes overlaid and a heat map of voxels engaged by at least 2 of the language processes (brighter voxels indicate more overlapping processes, up to a maximum of 5 shared processes in the brightest voxels).

In terms of the *task approach*, different tasks engage different parts of the language system to variable extents (Fig 2). Within the same patient, the left and the right hemispheres can both participate in - and rarely even be “dominant” for - different aspects of language (so-called “crossed dominance”).^43^ Consequently, **a combination of task contrasts assessing an array of language functions are generally advocated to establish laterality** at the lobe-level.^43–47^ Surveys^4,6,48,49^ indicate that epilepsy surgery and glioma-specialized centers typically assess language lateralization using at least two, and routinely 3 or more fMRI tasks.

In addition to the type of language task, laterality results are strongly influenced by specific paradigm choices regarding *control conditions*, i.e., what the language task is compared against.^42,50,51^ For example, comprehension tasks presented in the auditory modality offer poor lateralization if (the strongly bilateral^52^) acoustic processing is not accounted for. Similarly, multiple studies emphasize the importance of controlling for nonlinguistic aspects of language processing (e.g., using non-language visual or auditory decision tasks) to lateralize semantic processing in the temporal lobe.^50,53^ **Establishing regional laterality therefore requires careful consideration of both the task’s active and control conditions**.

General approaches to establish language laterality are presented in Box 2. Of note, **establishing lateralized representations of language functions does not exclude the possibility that an area of detected activation in the “non-dominant” hemisphere may be indispensable to language**.^54^ The “size” of an area of activation as detected by fMRI strongly depends on statistical analysis and thresholding of the data (section 3.3) and does not, *per se*, reflect its functional importance. Consequently, surgically-oriented applications of fMRI are more often concerned not only with establishing *which hemisphere* harbors language functions, but also with identifying *where they are located* and *what* language functions are most at risk.

#### 2.2.2. LOCALIZING LANGUAGE: FUNCTIONS OF CONCERN IN SPECIFIC BRAIN AREAS

Functional MRI efforts to localize language centers in relation to a focal lesion are intended to:

- predict the specific risks associated with the surgery,
- facilitate informed consent,
- provide an estimate of likely resectability / achievable resection extents,
- plan the surgical approach and / or
- guide the need for / use of Wada or intraoperative electrocortical stimulation mapping (ESM).

Among surveyed centers, almost 80% of neuro-oncology respondents use fMRI to localize language functions,^49^ with 88% requesting fMRI to help inform extents of resection.^68^ Some 44% of epilepsy centers already cautiously use fMRI to guide surgical margins.^69^ Suitability and limits of fMRI used for these purposes are difficult to evaluate. There is limited data systematically evaluating language outcomes, and high variability in how language is assessed both with fMRI and intra/peri-operatively. What is clear, however, is that clinical applications of fMRI to help decide ‘is this area safe to ‘cut’?’ generally require several carefully controlled tasks to isolate different aspects of language processing according to the structures most at risk in a given surgery.

**Fig 3.**
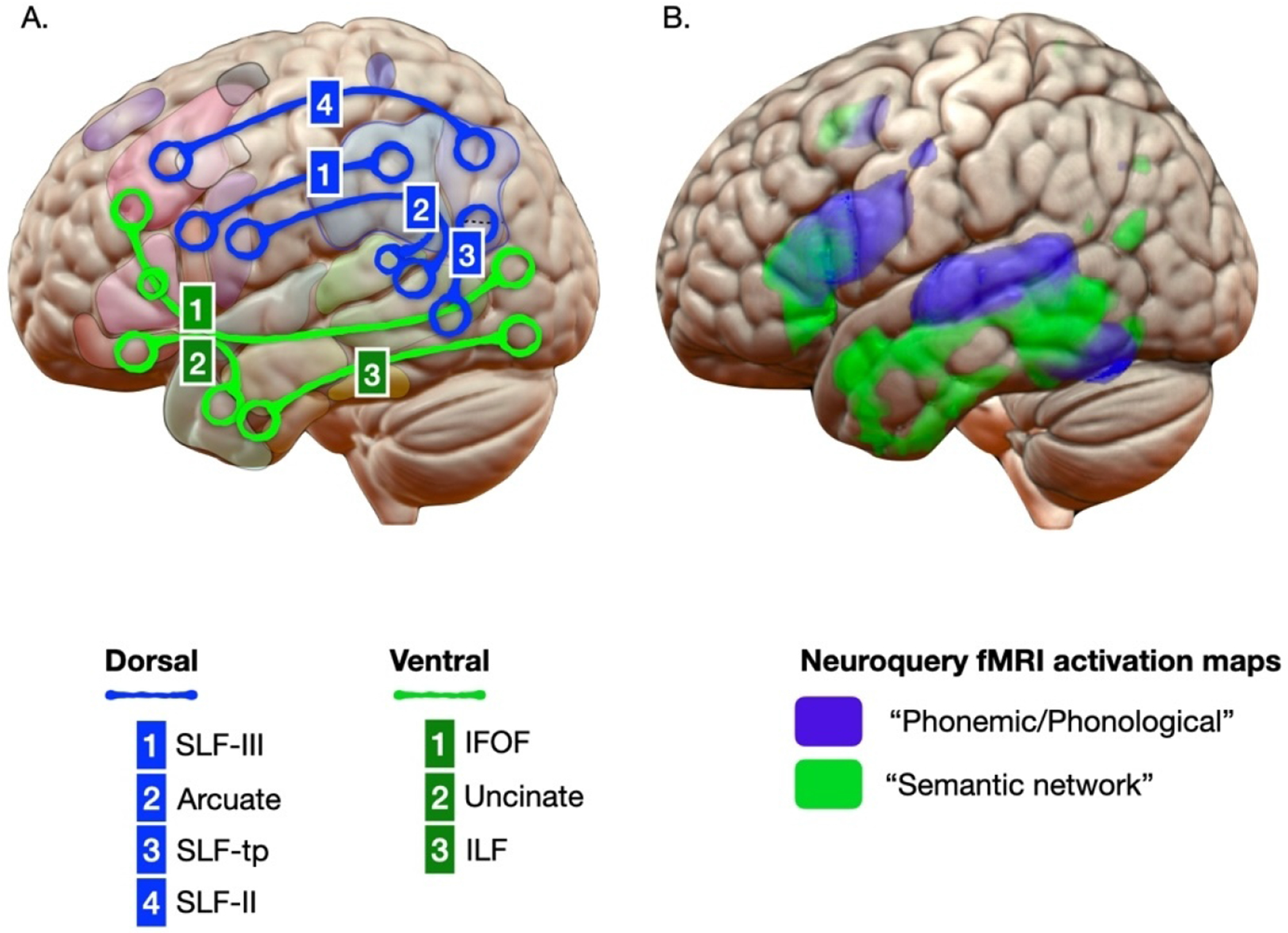
Theoretical ‘dual stream’ model of language processing A. Current prevalent model depicting at least two interacting streams of information processing that support phonological (blue, ‘dorsal pathway’) and semantic (green, ‘ventral pathway’, more bilateral) aspects of language function. Early studies proposed that information in these streams is conveyed by discrete sets of fiber bundles in the dorsal stream (predominantly the arcuate and branches of the superior longitudinal fasciculus) and in the ventral stream (predominantly the extreme capsule, i.e., inferior fronto-occipital fasciculus and / or uncinate). The dorsal and ventral streams largely (but not completely) distinguish between the ‘sound’ versus the ‘meaning’ of spoken language, and thus focus on auditory language processing. Nevertheless, there are also ventral parts of the brain involved in phonological processing (e.g., in the superior temporal sulcus) and dorsal parts of the brain involved in semantic processing (e.g., the angular gyrus). Recent extensions propose additional pathways that link visual information to the language system during reading and writing. B. Results of multivariate prediction-based meta-analysis of ~13,500 neuroimaging studies in Neuroquery (https://neuroquery.org/). Querying the search terms “phonological”/“phonology”/“phonetic” (blue) and “semantic”/“semantic network” (green) identifies patterns of cortical activation largely consistent with the dual stream model. The Neuroquery-derived z-score maps are shown thresholded at z=3.1 (corresponding to p<0.01). For detailed anatomical regions see Table 1.

Optimal fMRI tasks and task designs for language ‘localization’ remain an open question. In selecting an approach, recent reviews of widely used tasks can be found in references ^4,51,70–72^. Users could also consult large-scale neuroimaging database resources to observe the typical activation patterns of specific language tasks / processes (e.g., Figs 2, 3). Ultimately, however, fMRI task selection should draw on knowledge of the role of specific brain regions in essential aspects of language processing.^45,73–75^

Data supporting a role for certain brain structures in aspects of language have been widely replicated over the past 30 years.^73^ Questions surrounding language localization predominantly arise in the context of surgery involving:

- the inferior frontal gyrus (IFG),
- parts of the (especially posterior) middle frontal gyrus (MFG),
- posterior middle and superior temporal gyrus (MTG/pSTG) and sulcus (pSTS),
- inferior parietal lobule (IPL) and
- the mid-fusiform gyrus / basal occipitotemporal cortex.

Language-related deficits are also associated with surgery involving the supplementary motor area (SMA) and pre-SMA, but are mostly transient if the contralateral homologue can support this function^76^ and if the corpus callosum remains intact. Additional brain regions, including the temporal pole, the graphemic motor area (aka Exner’s) and Hopf’s area 55, contribute to language in ways that remain incompletely understood and are therefore challenging to ‘map’ (see full document).

In order to help preserve *function* after surgery, the aim of clinical fMRI is not to just activate a *specific brain region*, but to identify the *network* or assembly of brain regions engaged during *specific language processes*. This distinction is important for two reasons, which boil down to:

1. Several **neuronal populations contribute to more than one function**, and
2. ‘**Language’ is not a single unified behavior**. Brain regions that each perform more or less specialized computations likely combine in specific ways to support particular ‘language’ requirements.^77,78^

The ‘**core’ set of processes required for different aspects of language**^79^ includes a) *semantic access* (knowledge of concepts and meaning), b) *phonological representations* (the sound of words), c) *lexical* access (our store of learned vocabulary), d) *orthographic and graphemic knowledge* (visual word recognition and spelling), and e) *syntax* (knowledge of the rules governing word order and the functional roles of words in language). Speech generally requires *articulation* (planning, coordination and programming of speech), which is necessary but not specific to spoken language.^80,81^ At present, there remains uncertainty as to the level at which syntactic processing is supported by a neurally-distinct network in the brain,^82^ and about the neural substrates for temporal /syntactic order predictions (e.g.,^83–85^). Other cognitive processes also directly influence language performance.^86^

**Fig 4.**
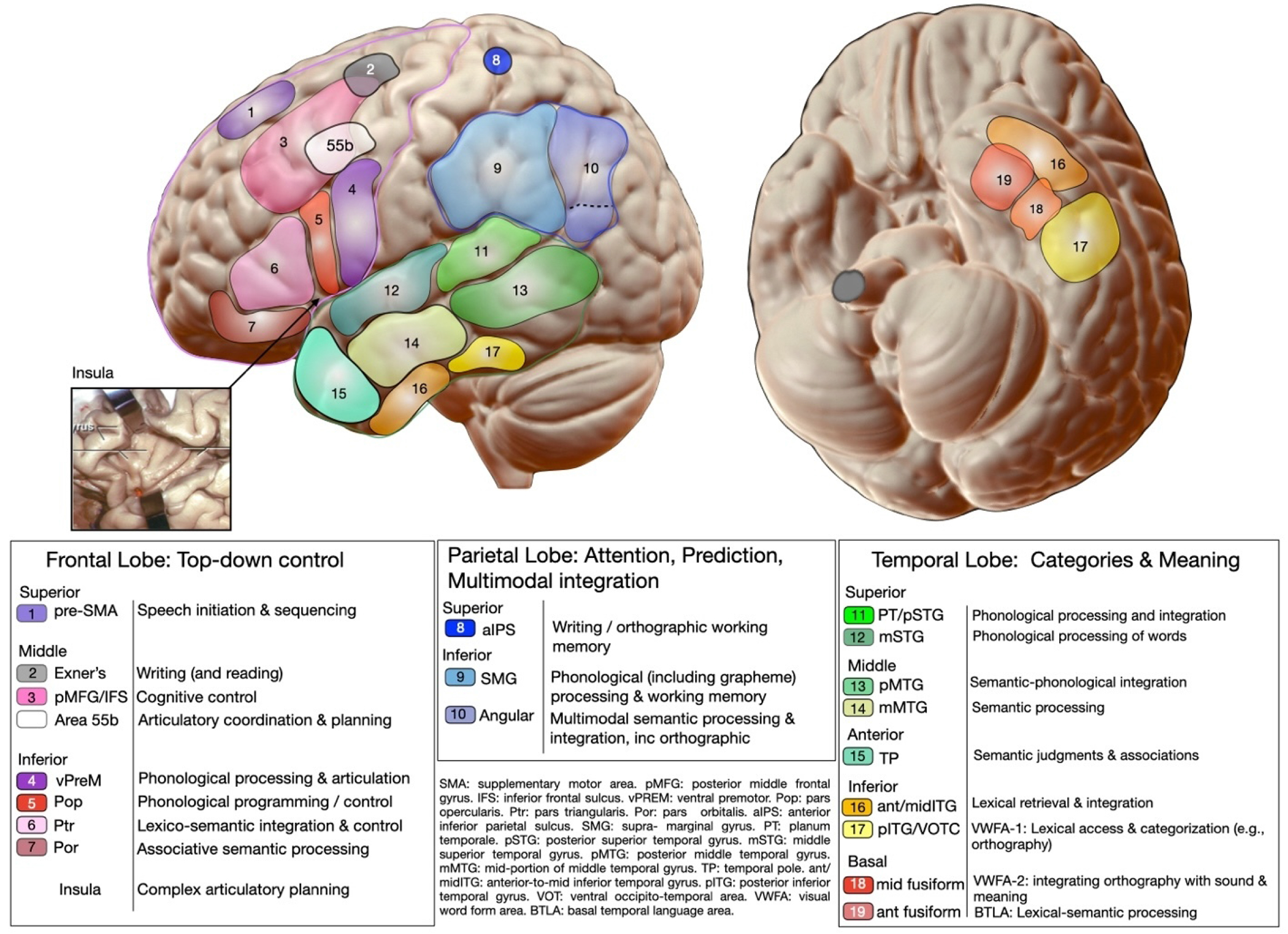
Language-related functional subdivisions of the neocortex Preferential roles of cortical sub-regions in language processing, based on a review of neuroimaging, intraoperative stimulation and lesion data (see Table 1). These summarized functions are neither absolute nor exclusive; many regions contribute to more than one language process, as well as to non-linguistic functions, and anatomical landmarks do not accurately correspond to functional boundaries, especially in patients. Instead, this figure highlights the higher *likelihood* of a given type of language function being represented in or near a given part of the brain, noting that both imaging and intraoperative findings are highly variable across individuals. Consequently, in a given patient undergoing surgery, it is relevant to consider a range of functions, allowing for the reasonable possibility that functions may have shifted (spatially due to mass effect, or as a result of functional reorganization). Subcortical structures including the basal ganglia and thalamus are known to contribute to language, but are not considered here due to the typical (resective) neurosurgical focus on functionality at the level of the neocortex (and underlying fiber tracts). Insula inset image adapted from.^100^

These ‘core’ language processes are often described in terms of (at least partially) discrete networks consisting of (at least partially) separate brain regions. The current dominant theory suggests two large-scale interacting systems^87,88^; one supporting conceptual (lexico-semantic) aspects of language and the other supporting phonological processing and speech.^89,90^ Fig 3 illustrates this dual stream model, presented alongside statistical maps of cortical brain regions activated during ‘semantic’ and ‘phonemic’ language processing according to predictive modeling of results from 13,450 neuroimaging studies in Neuroquery.^91^ Mapping specific language processes onto the dual-stream model is complicated by the fact that **most language tasks evoke the functions supported by both streams to varying degrees**. Additionally, many of the pioneers of language localization theories observed that **focal ‘deficits’** (or stimulation-induced disruptions in the case of Penfield) **likely reflect disruption to a wider language network**.^92^ Anomia, for example, is among the most frequently observed language deficits, but can result from disruption of several non-overlapping regions in the language-dominant hemisphere.^78,79^ That is, the *task* of naming is not ‘localizable’ *per se*.^93,94^ Nevertheless, converging data from stroke^93,95^ and resection outcome studies^96,97^ indicate that **some brain regions appear to contribute more critically to language than others**^78,79,93^ (Table 1). Allowing for our still-evolving understanding, Fig 4 summarizes the general approximations of core function-to-anatomy language mappings that are considered ‘reliable’ based on converging lesion, brain stimulation and imaging data (noting this is just one of different possible conceptualizations). Accordingly, the language processes most relevant to consider, and tasks commonly used to delineate them, are outlined in Table 2. When considering the tasks in Table 2, it’s essential to recognize variability in the effectiveness of theoretically appropriate language tasks. Some tasks demonstrate greater validity and reliability than others.^98,99^ Optimal tasks (i) reveal lateralization effectively, (ii) activate relevant language regions (validity) in a higher percentage of patients (reliability), and (iii) produce consistent maps across sessions (test-retest reliability). For example, semantic decision tasks generally yield stronger lateralization and more reliably activate frontal and temporal language regions than picture naming.^98^

### 2.3. DESIGN AND PARADIGM CONSIDERATIONS

#### 2.3.1. MAXIMIZING SENSITIVITY

Once the precise clinical question and target language processes have been identified, the sensitivity of fMRI will be influenced by how easily the language-related signal of interest can be distinguished from a comparison baseline signal (e.g., ‘rest’ or active ‘control’ conditions). When the aim is to capture the neural activity associated with a general task (naming, for example) but the specific responses (e.g., individual objects named) are not crucial, a simple **‘block design’ is favored** because it maximizes sensitivity to detect *average* BOLD responses.^101^ Using block designs, one or more language functions can be mapped in a relatively short experiment, making this the most prevalent design in clinical use,^4^ despite limitations, such as assuming a sustained BOLD response throughout each task block.

For some tasks, the objective is to isolate correct responses (e.g., only items named accurately). In these cases, capturing the neural response at precise timings is important, which requires ‘event-related’ analyses. The increased precision of event related designs, however, comes at the cost of reduced statistical power^101^ and requires longer acquisition times to reliably estimate overall neural responses, especially if there is a low(er) number of successful trials. Event-related designs are therefore not typically employed for clinical language mapping. However, further research into the potential benefits of ‘mixed’ block and event related designs is warranted.^102^

#### 2.3.2. ADVANTAGES OF A TASK PANEL APPROACH

Variations in task demands affect both within-hemisphere localization and lateralization. When the clinical objective is to establish language *laterality*, as mentioned, a standard panel of tasks can generate language maps in the language-dominant hemisphere that are superior (i.e., more ‘complete’) to those generated with a single task^45,103–105^. Because location and extent of activation for any language task depends as much on the chosen control condition as on the language task itself, it is highly recommended to conceptualize task protocols in terms of *task contrasts rather than isolated tasks*. When the clinical objective is to *localize* specific language-related processes around a surgical lesion, therefore, a **tailored task panel approach, contrasting different conditions, is typically needed to adequately probe all language functions at surgical risk**. Mapping distinct language processes *could* theoretically be achieved using a single paradigm containing multiple active conditions / task contrasts. However, there are advantages (e.g., minimizing movement) to employing short, targeted task contrasts, each separately assessing a given language process at risk. Further empirical data are needed to support the use of specific tasks over others for a given language process/region, but various options are listed in Table 2.

#### 2.3.3. OPTIMIZING SPECIFICITY

##### 2.3.3.1. MANY-TO-ONE PROCESSING

Neuroscientific and lesion-based evidence indicates that multiple cognitive processes can engage a single brain region; so-called ‘many-to-one mapping’.^106^ Choosing appropriate tasks – and task contrasts – that selectively engage individual brain regions is therefore a challenge. Within this constraint, general approaches are considered next.

##### 2.3.3.2. SELECTING TASK ‘CONTROL’ CONDITIONS

**Fig 5.**
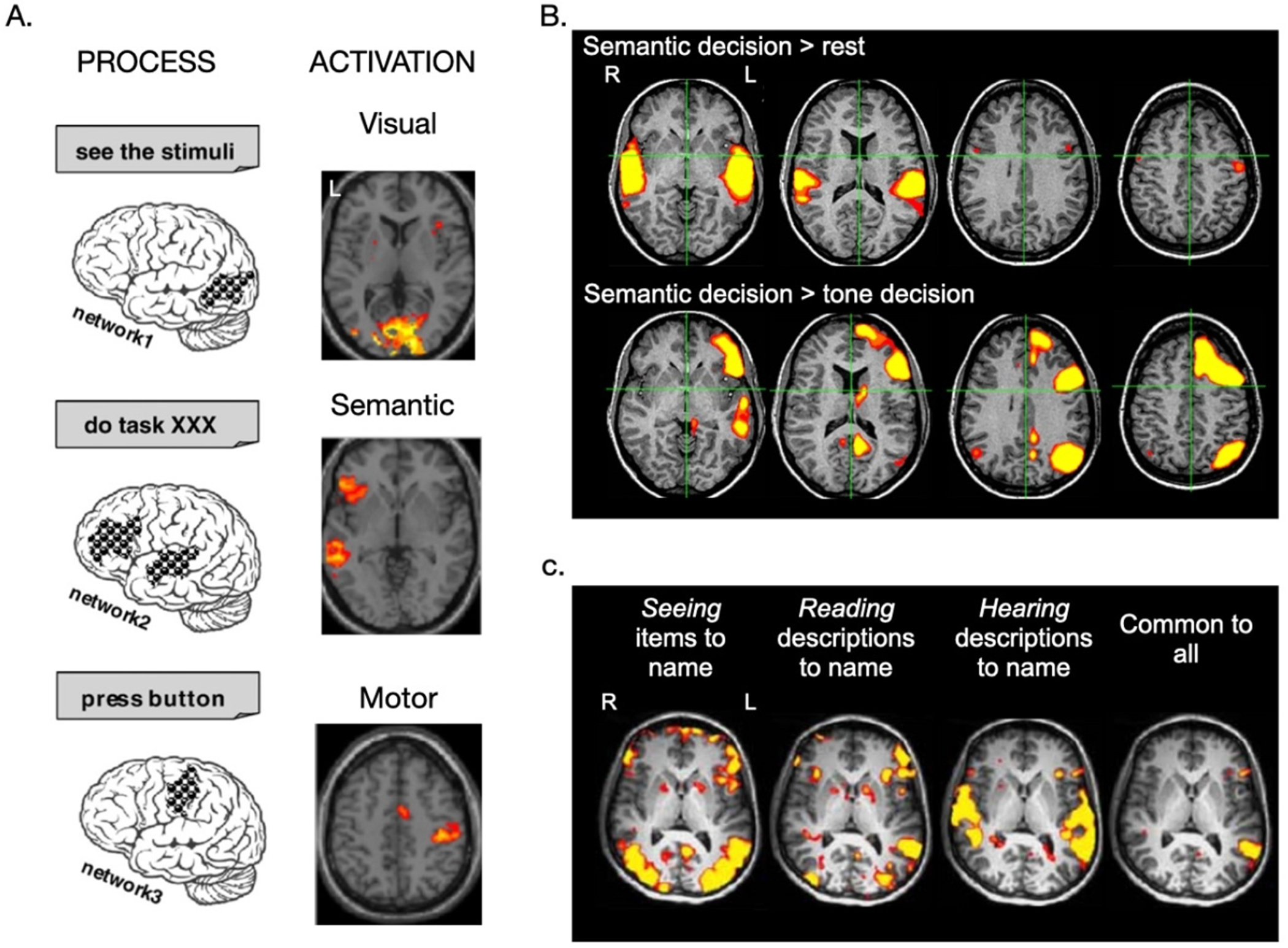
Effect of baseline condition and test modality on fMRI activation patterns a. Language activations maps almost invariably reflect a mixture of processes involved in the performance of a given task, often including sensory (e.g., visual) input unrelated to language, as well as motor (e.g., button press) or cognitive processes (e.g., decision-making) to generate a task-required response (adapted from^111^). b. Differential activation patterns during a semantic decision-making task^112^ (deciding whether aurally presented words are both “found in the USA” and “used by humans”) are found, depending on the choice of control condition: resting fixation, tone decision (deciding if auditory tone sequences contain two target tones or not). Note first the subtraction of almost all bilateral auditory sensory cortex activation (yellow-red color in top panel) when the comparison condition includes a control for low-level, non-linguistic auditory processing (bottom panel). Second, note the appearance of a large, left-lateralized frontal-temporal-parietal language network (bottom panel) when an active, non-linguistic control task is incorporated, which suppresses language processes that normally occur during ‘rest’. c. – Alternatively, taking a conjunction of different language tasks can identify core language regions. In this approach, a single task (naming) is chosen and repeated several times in different modalities (visual, auditory).

‘Resting’ remains a common baseline condition in clinical fMRI designs^4^ and is implemented in many commercial task-fMRI protocols that compare blocks when the task is ‘on’ versus ‘off’ (i.e., during rest). Pitfalls of using ‘rest’ as a comparison baseline have been highlighted previously.^107^ ‘Rest’ is an uncontrolled state^108^; important functions take place during ‘rest’, including memory consolidation^108^ and internal cognitive and linguistic processes,^109^ even when the ‘rest’ periods are very short (e.g., 3 seconds).^108^ Consequently, using ‘rest’ as the comparison in a language task limits sensitivity for detecting activation in language-related networks. Fig 5b shows the effect on a semantic decision language activation map just by varying the control condition from ‘rest’ to auditory tone decision. To increase sensitivity and specificity, carefully designed comparison conditions should aim to ‘control’ for aspects of task performance that co-occur with the language process of interest.^110^

**The choice of which control conditions to use is often a balance of *specificity* against *sensitivity* and *clinical feasibility***. Most commonly, the aim is to map complementary language processes using individual language tasks that will be analyzed separately. In this case, the choice of control condition in each task should reflect the desired level of precision in mapping each function (Table 2). Considerations are listed in Box 3.

#### 2.3.4. PRE-FMRI PRACTICE SESSION:

A recent survey indicated the most frequent reason for inconclusive language fMRI results was inadequate patient performance (47.3%),^6^ yet most participating centers spent <15 minutes on patient practice (55%) or gave no pre-scan instruction at all (14%). **The Working Group strongly recommends a practice session with the patient prior to fMRI,** to ensure they understand and are able (with appropriate modifications where possible) to perform the task as required, know when and how to make any required responses, and understand the problematic nature of head movements (further details: https://doi.org/10.31219/osf.io/r7u8p). For adults, preparation could consist of online training materials (e.g., stanfordhealthcare.org/fmri),^116^ supplemented with essential in-person practice. **For pediatric patients, virtual and in-person MRI simulator sessions** (e.g., https://www.bcchr.ca/3tmri/facilities/about-simulator) **are recommended where possible** to maximize success.

#### 2.3.5. MODIFICATIONS FOR PATIENTS WITH SPECIFIC IMPAIRMENTS

**Fig 6.**
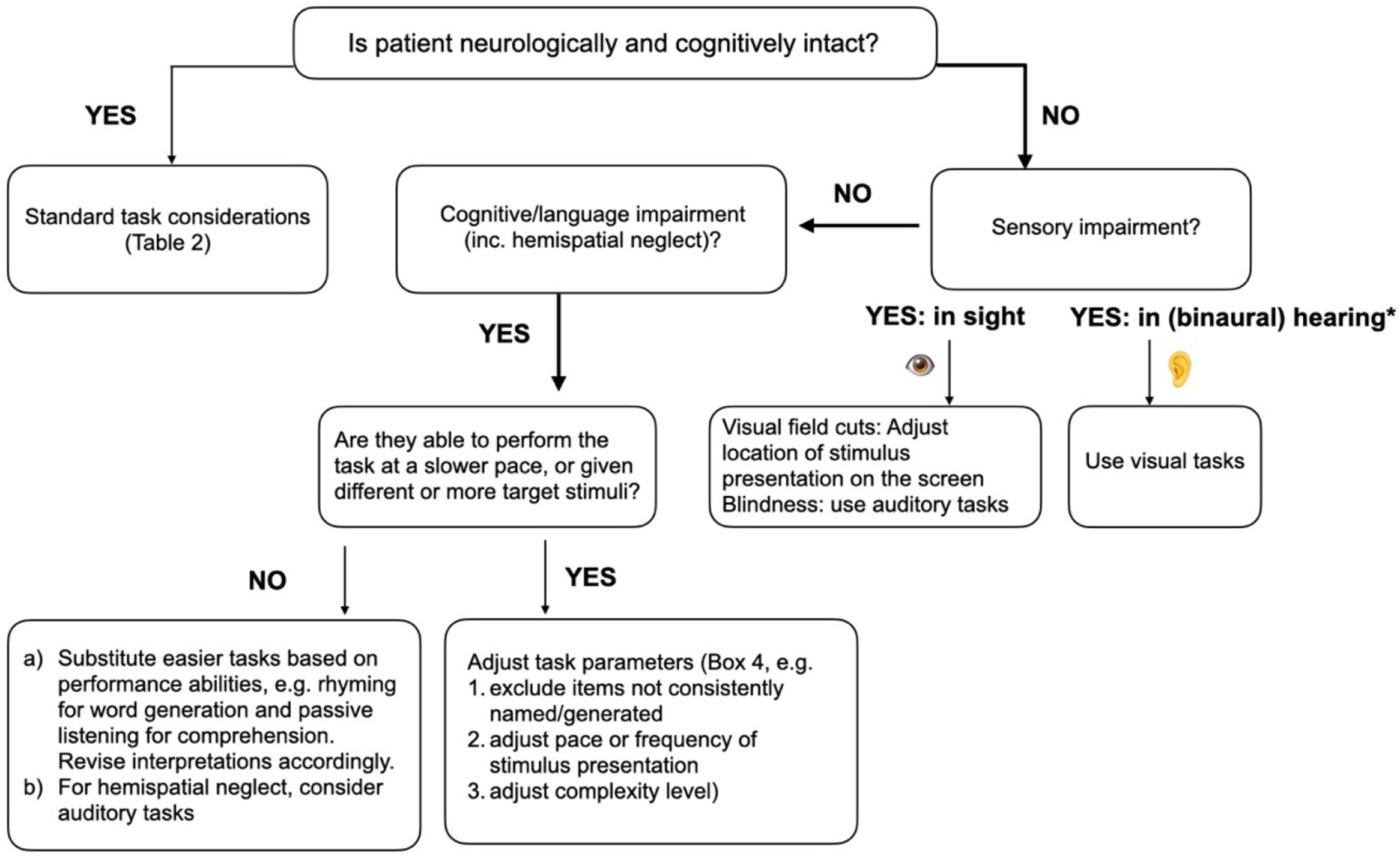
Proposed task adaptations for patients with sensory, cognitive or language impairments Proposed workflow to adapt fMRI language tasks for patients with specific deficits. For patients with concomitant hand weakness, if the task requires manual responses, possible adaptations include using an alternative hand, or capturing responses using alternative approaches (eye tracking if available, or using foot tapping to indicate which stimulus they wish to select, which are manually recorded by the person acquiring the fMRI scan). * Unilateral hearing deficits do not preclude patients from language mapping using auditory stimuli.

Specific considerations arise in populations presenting with various degrees of language deficit (Fig 6 and Box 4 of the full document).

##### 2.3.5.1. TASK MODALITY

Language task stimuli are most frequently presented visually.^4,7^ For patients with difficulties processing certain types of stimuli (e.g., written words in dyslexic or alexic individuals), or uncorrectable vision impairment, it may be necessary to change the modality of stimulus delivery (e.g., to auditory cues or picture cues) for language mapping to succeed (Appendix C).

##### 2.3.5.2. TASK SPEED & DIFFICULTY

Task difficulty influences fMRI activations in numerous language tasks.^114,117–121^ It is important to ensure a patient can perform the task over the entire session while keeping the task challenging enough to maintain continuous engagement (and minimize mind-wandering). However, when task performance becomes too difficult, the same regions can show less activity, indicating a relationship (often shaped like an inverted ‘U’) between task difficulty, cognitive effort, and BOLD response.^122,123^ Together, the data suggest that **tasks should be optimized for patient performance** - hard enough to require cognitive effort, not so difficult that the patient disengages altogether. A recommendation ***in patients with existing language impairments*** is to **maintain a balance between accuracy and motivation by targeting ~70-80% performance accuracy.**

Among our Working Group, some employ pediatric versions of a task in adult patients with aphasia or impaired speed of performance. Others have had good results with protocols developed for aphasic patients, such as Adaptive Language Mapping (ALM).^98,124^ ALM adjusts task difficulty on-line – based on performance – (https://aphasialab.org/alm/), using psychometric properties that have been quantified. ALM therefore offers an evidence-based approach, which, in one study, provided more robust laterality indices and stronger extents of activation than widely used clinical paradigms.^125^ When task modifications are not feasible on a per-patient basis (e.g., because of lack of options in certain commercial software packages), it becomes even more important to establish, through cognitive evaluations and pre-scan practice, that patients can perform adequately.

#### 2.3.6. CONSIDERATIONS FOR PEDIATRIC PATIENTS

A study of over 400 language fMRI scans in clinical child populations indicated an overall promising success rate of fMRI, but a higher rate (~15.8%) of ‘failed’ scans when compared to typically developing children.^126^ Predictably, ‘failed’ scans increase with lower age^127^ and is driven primarily by head motion, which can be mitigated. ^128,129^. Scan failures are also attributed to some children falling asleep.^126^ These findings emphasize that **children may benefit particularly from task modifications that a) sustain attention and effort** using several (short) tasks, **and b) ensure a minimum number of successful responses**.^130^ Clinical fMRI is generally more successful in children > 7 years old.^126^ It **is recommended to use at least 3-4 short tasks**,^44,131^ partly because head motion increases with every additional minute of acquisition.^132^ Activation procedures should be **individually tailored according to performance level** (as for adults), but also according to **developmental stage**.^44,133^ This is because non-linguistic ‘difficulty’-related contralateral activations likely drive an increase in apparent ‘bilaterality’ in some fMRI tasks relative to language lateralization results from Wada testing.^131^ **Selecting an active comparison condition** is especially beneficial to maintain attention and engagement in younger children who are less able to comply with the requirements of ‘resting fixation’^134^ and/or children with neurodevelopmental and behavioral disorders (considered in^135^). Making the scanning environment easier for children, through mock sessions or training, can be beneficial for increasing compliance with task-based fMRI. Strategies such as using favorite toys or playful scenarios (e.g., imaging the MRI as a spacecraft) may also help engage the child and reduce anxiety. These considerations highlight the need for flexibility and case-specific strategies in surgical planning.

A very wide range of tasks and task contrasts has been described for pediatric language fMRI. Data showing probabilistic activation maps or comparisons between these tasks in terms of lateralization or activation pattern in children remain lacking. When considering an optimal battery of pediatric language fMRI tasks, one solution is to employ a different panel of tasks best suited to specific age ranges. The main drawback of such an approach is that the interpretation of results between different age groups, and, importantly, in the same child over time, becomes challenging.^136^ An alternative solution is to adopt a single battery of tasks, in which each task aims to engage a specific language process (e.g., phonology, semantic retrieval, …), using parallel versions that vary in difficulty level. Specific task considerations are discussed in the full document: https://doi.org/10.31219/osf.io/r7u8p. Further development is advocated to establish age-appropriate naming stimuli and norms in pediatric populations.^137^ An additional consideration is that pediatric language mapping may be influenced by higher brain metabolism affecting the measured BOLD signal.

#### 2.3.7. BI- AND MULTILINGUAL PATIENTS

In bilingual neurosurgical patients, in addition to brain regions that are shared, separate areas of cortex uniquely subserve the primary versus additional languages^138^ (see full document). **Language fMRI mapping should prioritize the primary language, but should ideally include all languages used by a patient in their everyday life**.

Certain tasks, such as silent object naming, can be administered in multiple languages without the need to modify stimuli. However, cultural differences in familiarity with certain stimuli may affect performance. The difficulty of other tasks, such as phonemic fluency, depends on the frequency of individual letters in different languages, which should be chosen according to available normative data (i.e., appropriate ‘difficulty’ level) in each language. Several common paradigms are available in multiple languages as part of some commercial packages, or freely from research groups (www.cogneuro.net/hbm2017^75^). Several participants of the Working Group employ **parallel language versions of tasks,** especially for reading and comprehension tasks.

Over and above possible differences in the brain regions supporting language production, speakers of multiple languages make use of a distributed ‘control’ network.^139–141^ Damage to this network can result in uncontrolled fixation to a single language^142,143^ or mixing/switching of languages without aphasic symptoms.^144,145^ Therefore, **a language switching task is useful to consider when undertaking language fMRI mapping in bilingual patients**.^139^ Perhaps the simplest would be word translation (present word in one language and request a response in another language), or alternating language blocks (e.g., using a country flag to indicate the target language^146^).

## 3. PART 2. ACQUISITION AND ANALYSIS OF CLINICAL FMRI DATA

### 3.1. SKILLS & TRAINING

While fMRI is sometimes considered a standard neuroradiological exam, it is not. Clinical language fMRI is a *form of cognitive assessment* **and requires case-by-case input from experts in multiple domains**.^9^ **Professionals with extensive multidisciplinary training in clinical fMRI, or pairs with complementary training are needed for optimal patient care** (see https://doi.org/10.31219/osf.io/r7u8p). It is beneficial if the same specialist practitioner can accompany the patient through the multiple stages of the process (i.e., pre-op and follow-up evaluations, fMRI, and intra-operative assessment if performed).

### 3.2. DATA ACQUISITION

Technical aspects of MRI data acquisition and processing impact on the success and utility of clinical fMRI (summarized in Box 5 of the full document, here: https://doi.org/10.31219/osf.io/r7u8p). Among the most critical recommendations is to **monitor the success and quality of fMRI exams in real time and seek feedback from patients after every fMRI run**, so that any apparent problems (e.g., motion or inadequate task compliance) can be addressed.

**Fig 7.**
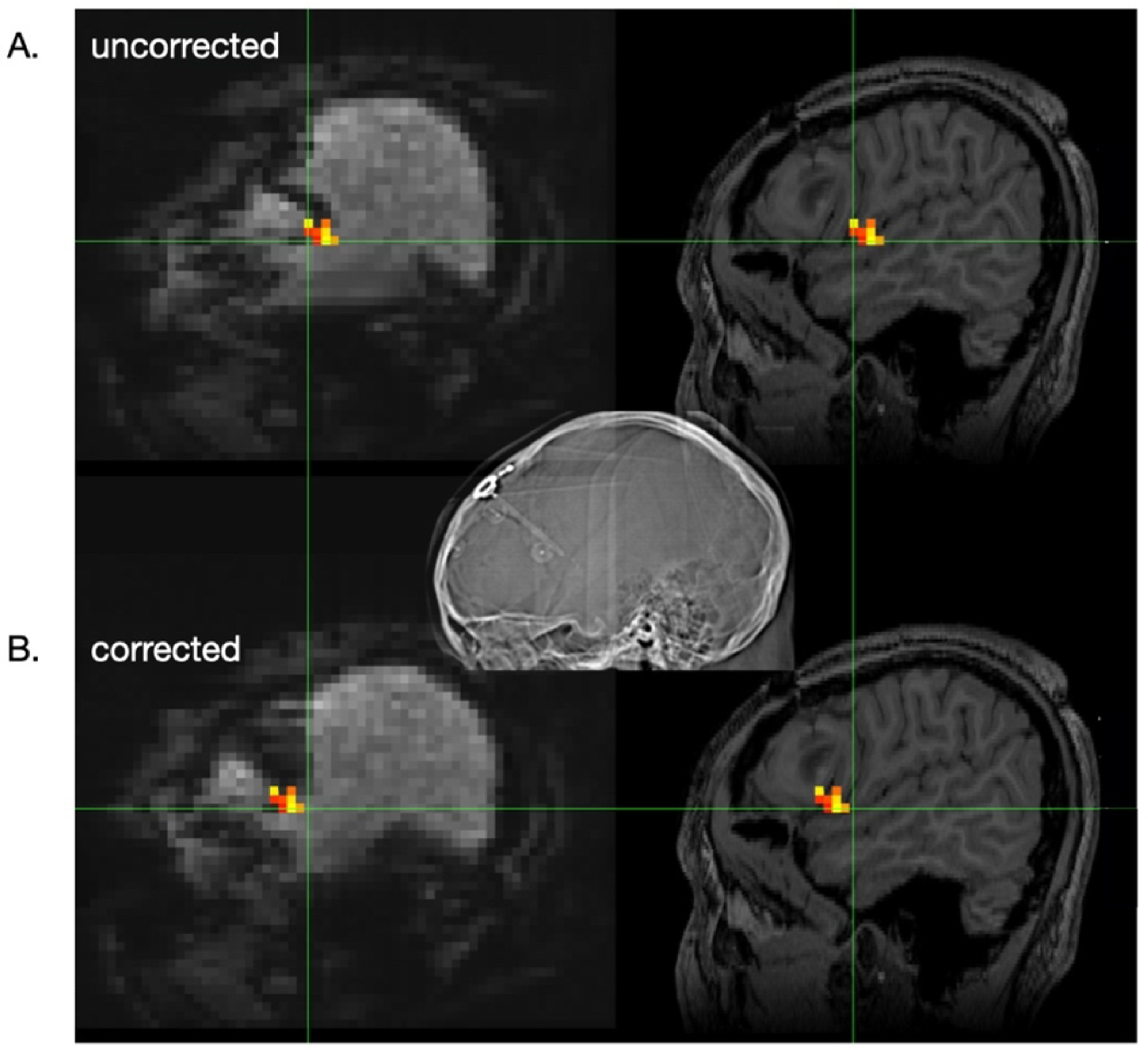
Displacement of fMRI task-detected activations due to echo planar imaging distortions A. Gradient-echo echo-planar images (GE-EPI) - the most common fMRI sequence - are most often acquired with a phase encode direction from anterior to posterior (as illustrated here), resulting in typical anatomical deflection of the brain inwards (i.e., in the posterior direction, top left image, see distorted image anatomy at the anterior frontal and temporal lobes). Areas of signal loss (e.g., around the paranasal sinuses and temporal bones, or from metallic abrasions/craniofix of previous trepanations, central X-ray inset revealing the previous osteoplastic trepanation and Rickham catheter with Ommaya reservoir) cannot be recovered, but several methods exist to ‘correct’ for anatomical distortions. If fMRI task activations are overlaid onto the (non-distorted) anatomical scan for surgical neuronavigation, these EPI distortions, if not corrected for, can create large mismatches in where the activations appear to be located (compare location of the fMRI task activations and crosshair in the distortion-uncorrected top right image against location of the fMRI activations / crosshair in the distortion-corrected bottom right image). B. Illustration of the effect of distortion correction on the localization of language task-related fMRI activation results. Note the location of task-related activation relative to the lesion is substantially different: the uncorrected images imply activation is located at least 1 gyrus backward from the lesion, whereas the appropriately corrected images show intraoperative stimulation confirmed language task-related activation directly inferior to the lesion, in this case a left frontal ganglioglioma. Note that distortions were particularly pronounced due to prior surgery with craniofix and Ommaya reservoir implantation.

Performing task fMRI requires dedicated hardware. A combination of visual and auditory tasks allows for more comprehensive mapping of language networks than tasks delivered in only one modality (e.g.,^52,53,62,147^), as also reported for intraoperative stimulation mapping.^148^ Dedicated equipment and sequence properties are considered in the full document. It is recommended that additional **field mapping sequences** are acquired for subsequent correction of EPI distortions and **accurate alignment to** non-EPI (e.g., **T1) data** (Fig 7).

As part of fMRI scans, complementary acquisition of **diffusion** (tensor, or ideally more advanced) **imaging to reconstruct fiber tracts involved in speech and language processing is recommended** (Appendix D). Cerebrovascular reactivity mapping can additionally be considered (Appendix F).

### 3.3. analysis

Unlike structural MRI, it is not the acquired fMRI-BOLD images themselves, but the statistical results generated from them upon which clinical interpretations are based. Choices in processing and statistical analysis can fundamentally impact results, revealing or obscuring task-related signal. Comprehensive reviews have detailed imaging analysis ‘best practices’ for the general neuroimaging community.^13,149,150^ However, **decisions relevant to analyzing individual patient data differ in important ways from guidelines that apply to group analyses**. A few select processing choices that particularly impact on the interpretability of *single-subject clinical fMRI results* are considered below (summary in Table 3 of the full document). The single most important recommendation is to **undertake quality control at every step**.

#### 3.3.1. HEAD MOTION AND MOTION CORRECTION

Language tasks, especially those involving overt speech, are particularly prone to stimulus- or task-correlated motion. Objective criteria for how much head motion is “too much” cannot be easily established, but in the worst case, head motion can make brain activation uninterpretable. **The best and most effective approach for dealing with head motion is prevention,** often readily achievable through careful patient preparation and real-time inspection of the images so that scans can be repeated, if required, before the patient leaves the scanner. Various strategies to ‘correct’ head motion exist^151^ (Appendix E) and **practices vary widely.** This group’s consensus is that **neither prospective nor retrospective motion correction should be assumed to fix all issues arising from head motion. Subject head motion should be assessed in each fMRI scan through a variety of means**. This includes inspection of the raw images and retrospective motion correction / realignment plots, as well as performing multiple analyses to compare the effects of different motion correction choices on the resulting activation maps.

#### 3.3.2. BRAIN EXTRACTION AND STATISTICAL MASK GENERATION

Some data pre-processing pipelines remove non-brain tissue (eyes, orbits, skull and dura) from the images. This step can have the unintended effect of also removing low-signal lesions and their perilesional areas.^152^ If brain extraction/mask generation is part of the initial processing pathway, it is crucial to **verify that brain extraction has not also excluded the pathological lesion and the area around it from statistical analysis** and, if necessary, edit or replace the brain mask.

#### 3.3.3. SPATIAL SMOOTHING

**Fig 8.**
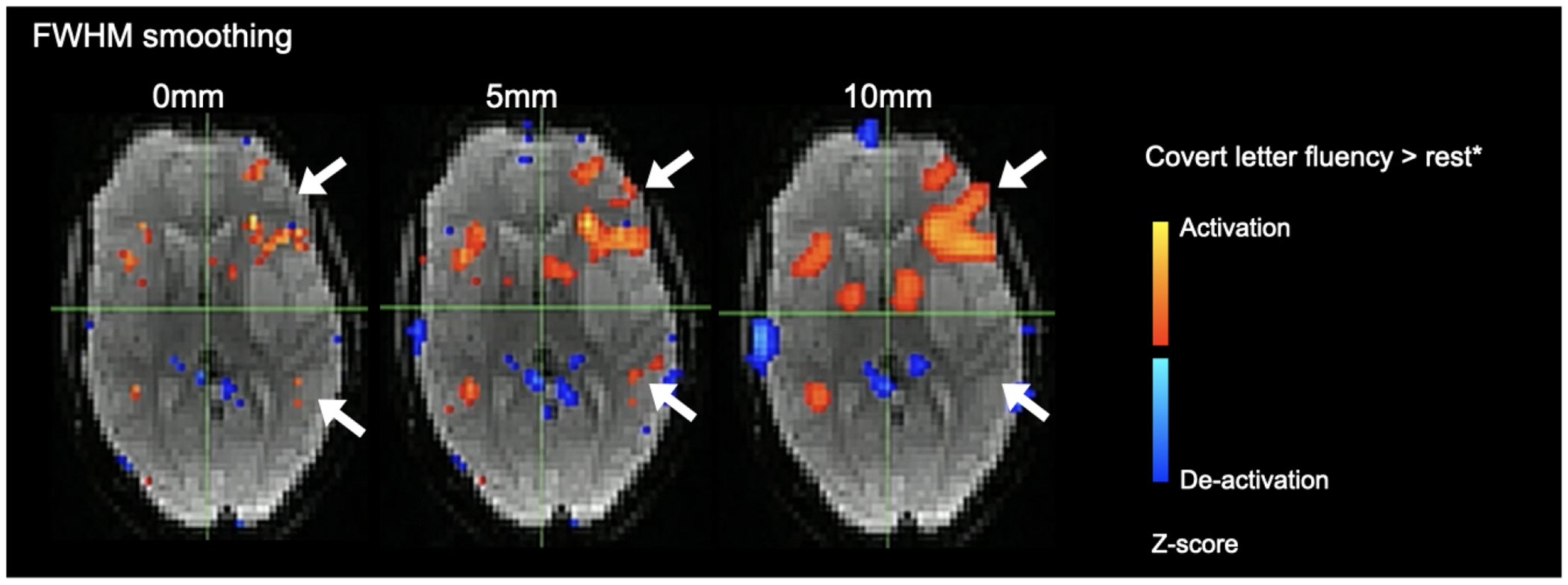
Effect of spatial soothing on statistical activation maps Effect of varying spatial smoothing on an example word generation language activation map, acquired at a voxel resolution of 2x2x2mm, illustrated in a patient with a left fronto-insular glioma. The amount of smoothing was varied from 0 to 10mm while keeping all other analysis steps constant. The resulting spatial maps (each presented at the same statistical threshold) show substantial influence of the choice of smoothing on the spatial extent and foci of activation (e.g., white arrows). * Resting fixation is not generally recommended as a task contrast; it is used here merely to illustrate the effects of spatial smoothing on activation maps in general (irrespective of task/contrast). FWHM: Full width at half maximum.

Spatial smoothing can improve SNR and statistical power in fMRI. However, this is only true if the extent of the smoothing is less than the size of the activations; large amounts of smoothing reduce the ability to detect smaller activations (Fig 8) and can spatially displace their focus.^153^ To retain spatial specificity in activation maps for presurgical clinical fMRI applications, the consensus of this Working Group is to **avoid or minimize smoothing, using no more than 1 to 2 times the voxel dimensions**,^154^ **up to a maximum of 5mm** isotropic FWHM (for a 2 to 3mm voxel dimension), **but ideally <4mm**. This recommendation minimizes the chances of blurring together noncontiguous cortical speech processing areas (e.g., for word production versus word hearing) identified 4mm apart using high density subdural electrode grids.^155^ This approach furthermore aims to balance smoothing as little as possible while retaining conspicuity of activations of interest (which can be more difficult to identify in unsmoothed data).

#### 3.3.4. REGISTRATION OF CLINICAL FMRI AND HIGH-RESOLUTION STRUCTURAL IMAGES

**Whenever fMRI results are overlaid onto an anatomical scan, it is important to identify and communicate any limitations in the underlying fMRI data,** such as areas of susceptibility-related signal loss in fMRI (often affecting the inferolateral temporal lobe) which can lead to false-negative interpretations (e.g., in relation to a visual naming or reading task). Such misinterpretations can usually be avoided by **first inspecting results in the original fMRI data space**.

#### 3.3.5. DATA ANALYSIS AND STATISTICAL INFERENCE

Meaningful clinical interpretation of fMRI data requires pragmatic knowledge of the advantages and pitfalls associated with the processing steps used for generating fMRI results.

Several commercial analysis packages exist for clinical fMRI analysis. These typically do not offer the user (m)any options to vary (or verify) parameters relating to statistical inference and thresholding. Therefore, in the wider community and among this Working Group, freely available research packages are commonly used, alongside clinically-licensed ones, for the added benefits the former provide.^4,6^ However, **fMRI analysis packages developed for research use are typically not approved for clinical use and are optimized for a different use**. An appreciation of the rationale behind analysis settings is important to understand why it **is suboptimal to apply default recommended settings in some *research* fMRI analyses to *clinical* applications.**

##### 3.3.5.1. HYPOTHESIS TESTING

The most common approach to analyze fMRI data - including clinical fMRI - is hypothesis-driven general linear model (GLM) fitting. A simple GLM implementation is available on many scanners and can be used to monitor fMRI scans in real-time. Real-time monitoring of activation maps while the patient is on the table can ensure that the collected data will be high yield and contain useful information for producing final fMRI activation maps (or allow the scan to be repeated with renewed instructions). Some members of this Working Group routinely supplement GLM-based analyses with data-driven spatial independent component analysis (ICA), which does not impose the same assumptions as the GLM (Appendix E).

##### 3.3.5.2. EFFECT SIZE MAPS, STATISTICAL THRESHOLDING AND INFERENCE

Statistical testing and inference (including thresholding) constitute a major challenge for clinical fMRI. Thresholding turns fMRI results into maps of activation (or no activation), which guides the neurosurgical decision as to what tissue is potentially resectable (subject to intra-operative confirmation^156^) or should be avoided. However, **currently, there is no standard or common approach that guides how to best threshold statistical fMRI maps for clinical purposes**.

The first key step is to decide–among all the signals detected during the fMRI scan–which of the brain voxels or clusters of voxels show a signal that we are confident is related to the task (see Appendix E for elaboration on this critical topic). Selecting the appropriate statistical criteria to answer this question depends on assumptions about the (temporal onset, magnitude and extent of) fMRI signal measured, as well as choices about when we have statistical confidence in the detected activations. A false positive (FP) result – indicating more areas of activation than are truly there – could unnecessarily prevent surgery or maximal resection. Conversely, false negative (FN) results – where areas of language activity are erroneously ‘missed’ due to statistical choices – generally pose the highest concern in pre-surgical applications of fMRI. **A false negative result in fMRI maps that is not interpreted with appropriate caution might result in surgical removal of an area of cortex that is crucial for language abilities**. Consequently, choices in the statistical analysis of clinical fMRI data *should* be balanced towards avoiding FN (i.e., type II) errors. **However, most widely used fMRI analysis packages are** based on classical statistical inference that controls for FP rates, and are therefore **not optimized (nor fully adequate) for clinical fMRI**. Alternative statistical approaches are available that aim to control the balance between FNs and FPs (e.g.,^157–161^). However, these require broader validation. This working group particularly encourages the collection and pooling of such data / analyses across sites.

Once a task activation map has been generated, a second decision arises *whether* and *how* to threshold the resulting image. The magnitude of fMRI activation can vary substantially across individuals because of the effects of the lesion on the fMRI signal, different levels of performance/impairment,^162^ head motion and certain medications,^163^ among others. Appropriate thresholds differ for different individuals^164^ and **choices need to be tailored to each patient**. **Our group recommends inspecting a range of statistical thresholds as well as the un-thresholded results** both for every activation map, as well as task de-activations, in and around areas of interest for presurgical mapping. As a general principle, we recommend that established “institutional approaches” are reported clearly and evaluated rigorously against all available clinical data, most especially post-operative language outcomes.

#### 3.3.6. CALCULATING LATERALITY

Best practices in calculating laterality both in general and for clinical practice are still undefined.^47^ From a practical (analysis) perspective, the outcome of language laterality calculations with fMRI can change depending on the statistical threshold employed.^165,166^ Using a single fixed statistical threshold (i.e., not tailored to the individual) increases a risk of suboptimal or inaccurate assessment of fMRI language lateralization,^167–169^ especially when cognitive deficits or pathological features may reduce the overall magnitude (or height) of BOLD fMRI signals.^165^ Since language laterality is a continuum,^170,171^ the question of how to determine laterality based on fMRI is an important methodological challenge.

Many methodological variations to analyze fMRI language laterality have been proposed and evaluated against the Wada test (e.g.,^21,44,168,172^). However, the Wada test is itself not infallible (see^41,173,174^ and references therein). Consequently, the “ground truth” of language organization in the individual patient may not be absolutely known, with the result that the superiority of any one method of calculating a fMRI laterality index (LI) over the others remains undetermined. **If LIs are generated, it is generally undisputed that LIs calculated at a single standard statistical threshold are not adequate**,^47^ especially for clinical use. Additionally, a single ‘global’ LI is likely to be misleading in rare patients who have mixed dominance (e.g., anterior and posterior language areas in different hemispheres). In such cases, at any given threshold, laterality indices may be biased towards the non-affected hemisphere, limiting their utility. Consequently, when the clinical question is purely to establish relative lateralization to inform discussions around surgery, our recommendations are:

1. To **include an appropriate range of tasks (and carefully consider task contrasts)** to establish lateralization for the components of speech and language of greatest surgical relevance.
2. If LIs are calculated, **visualize a range of LIs tailored to the patient’s** levels (i.e., signal magnitude) of **activation, or** employ an LI calculation method that produces **a weighted average across a range of thresholds** (e.g.,^175^, see other approaches described in^47^).
3. **Consider LIs from different language-related regions (not just the frontal lobe),** but excluding regions involved in sensory (e.g., visual or auditory) or cognitive processes not specific to language (e.g., frontal eye fields, dorsolateral prefrontal cortex, anterior cingulate cortex).^46^
4. **If the fMRI results are categorized** (i.e., into ‘left-lateralized’, ‘right-lateralized’ or ‘mixed/bilateral’), **indicate how these categories were defined** (e.g., based on arbitrary LI cut-offs, or by comparison to specific population norms). Caution is advised in how atypical laterality is interpreted, noting high variations among experts in the meaning of ‘bilateral/mixed’ and ‘atypical’ dominance.^47^ Some clinicians might conclude from a non-nuanced fMRI report of a ‘mixed’ or ‘bilateral’ LI that surgery on the left hemisphere is without risk (e.g., interpreting this result as showing that both hemispheres are functionally equal), whereas *such an interpretation is likely neither intended nor indicated*, especially in patients with existing language impairments.

### 3.4. REPORTING & INTERPRETING FMRI RESULTS

**Images are powerful**. Great care should be taken to minimize misinterpretation, especially in terms of an apparent absence of activation near a susceptibility artifact, or when superimposing fMRI data onto structural images in which areas of artifact may be less prominent / invisible. Specific emphasis should be placed on limitations of a particular exam (e.g., regarding patient head motion, impairments, etc.). It is the opinion of this Working Group **that if the person interpreting the data does not believe the report to be valid, no images should be generated at all**.

fMRI reporting best practices have been proposed for the neuroscience community^13^ and complement guidelines for BOLD-fMRI dictation from the ASFNR (https://www.asfnr.org/wp-content/uploads/BOLD-fMRI-Dictation-Guidelines.pdf). Our Working Group recommends specific additions relating to the description of tasks and analysis methods used (Appendix G). Additional oral presentation of the fMRI findings, i.e., at surgical team meetings, offers valuable opportunities to highlight confidence and specific limitations of the results in an individual patient’s scan.

Importantly, while some surgical programs report using precise – but different – distance limits from fMRI activations to guide surgical margins,^69^ given skilled clinicians, extensive experience, and cautious interpretation, this approach is *not likely to be accurate* in most (if not all) cases, because **distance calculations in fMRI are intrinsically related to preprocessing steps** (especially smoothing) and statistical choices made by the user, rather than reflecting true functional boundaries.

## 4. DISCUSSION

### 4.1. SUMMARY

This document presents a summary of best practice recommendations from the OHBM Working Group on clinical language fMRI mapping. Our approach to generating these recommendations was based on the consensus and experience of this multidisciplinary committee, supplemented with the weight of available evidence behind clinical decision-making. Previous groups have reviewed a different range of clinical fMRI language applications and found different levels of validation for them. For example, the level of validation was higher for predicting language outcomes based on fMRI laterality assessments than for preventing deficits with MRI localization and tailored surgery, at least in epilepsy.^5^ Here, we further consider language tasks and task designs optimized according to specific clinical objectives. In doing so, we focus on specific patient populations (e.g., adult/pediatric) and possible modifications for their particular needs (for expanded considerations, please see https://doi.org/10.31219/osf.io/r7u8p). Putting forward recommendations based on the strongest available data remains challenging due to the absence of randomized controlled trials of language fMRI applied to most surgical populations. A second obstacle is the sparsity of studies conducting head-to-head comparisons of different tasks, and their performance when predicting post-operative language outcomes. There remains, therefore, a long way to go. Given these challenges, we place emphasis on *language processes* that should inform the selection of task fMRI, focusing on data that survive meta-analysis (predominantly for language lateralization) and converging lines of evidence from research neuroscience studies and surgical lesion outcome data (in the case of localization). We put forward practical guidance, based on state-of-the-art in neuroimaging science, for all aspects of clinical fMRI in relation to acquisition and analysis of individual patient data. Of course, our understanding of the basis of language in the brain, as well as technical implementations, continue to evolve. These recommendations do not aim to be final or prescriptive. Instead, our objective is to offer practical steps and guidelines for generating a shared knowledge base and collaborations which promote consistency in how fMRI language mapping is performed. In this way, this document aims to improve minimum standards and facilitate the objective assessment and quantification of the benefits, efficacy and limits of high-quality clinical fMRI. Achieving the latter requires thoughtful application to ensure that fMRI is:

- performed in a multidisciplinary manner, based on careful definition of individual patient characteristics and performance abilities
- backed by converging neuroscientific and clinical data
- follows best practices in data acquisition, processing and analysis
- is interpreted and used by the operating neurosurgeon based on interdisciplinary consultation
- systematically evaluated against clinical outcomes to determine added value for minimizing language-related risks

In this process, a clear need was identified for the wider reporting of individual groups’ experience in relation to fMRI, including relevant factors such as amount of ESM mapping required and duration of awake surgery based on fMRI predictions, and crucially, language outcomes. Some identified avenues for targeted developments and research, needed for language fMRI to substantiate and improve its clinical utility, propagation and availability, are detailed in Box 6.

### 4.2. WORKING GROUP’S POSITION ON THE UTILITY OF CLINICAL FMRI

An estimated 30-50% of European neurooncological centers^49,176^ and most (>90%) epilepsy surgical programs worldwide^69^ employ fMRI. A recent survey of US pediatric epilepsy surgery centers in the Pediatric Epilepsy Research Foundation Surgery workgroup found that 100% of surveyed sites were using fMRI for functional mapping, and more than 80 % also perform language mapping with implanted stereo-EEG-based ESM as an emerging approach to complement fMRI.^177^ Yet, justified uncertainty persists around fMRI’s ability to localize functions with the precision required for surgical planning. There are inherent constraints on the spatial precision that fMRI can achieve based on the limitations of the underlying BOLD contrast.^178^ Frequently cited confounds include the difficulty dissociating task-associated from language-essential neural activity with fMRI, and consequent variable sensitivity and specificity of fMRI relative to ‘virtual lesioning’ techniques.^179^

Evidence from epilepsy surgery candidates demonstrates the suitability of using fMRI as a surrogate for Wada testing to establish language dominance. As long as care was taken with task selection and design, fMRI has shown ability to predict postsurgical language outcomes to some degree. Measuring the effectiveness of language mapping in these scenarios remains difficult, perhaps impossible, in clinical studies in which the maps are used to adjust the surgical procedure, since there is no comparison showing what the outcome would have been without fMRI. As a result, the literature consists of mainly uncontrolled observations and the occasional comparison to historical control groups.

Cases of failure to prevent language declines likely exist, according to a recent clinical survey reporting the relatively common practice of resecting fMRI ‘activations’.^69^ Conversely, 17% of epilepsy surgical programs reported one or more cases where all language fMRI-positive activation was *preserved*, but a patient still suffered post-operative language decline (noting possible contributions of subcortical tract damage). None of these had been published. According to a recent survey on post-surgical care practices in Europe, not all neurosurgical centers refer each patient for neuropsychological assessments to evaluate language performance / outcomes. Some patient groups are particularly unlikely to undergo language evaluation; only 3% of patients with high-grade gliomas were offered language assessments after brain surgery, in comparison to 30% of individuals with low-grade gliomas.^180^ Practical challenges clearly arise in the neuropsychological scheduling and follow-up of patients who are on a rapid treatment pathway, or are due to undergo post-surgical adjuvant treatment. This appears to be less problematic in an epilepsy setting, where 70% of patients receive follow up.^69^ However, **systematic, longitudinal outcome reporting is much needed to help inform what clinical questions fMRI is suited, or not suited to answer, and potential reasons for fMRI failures, if these are encountered.**

In the opinion of this Working Group and with growing evidence of its predictive power, language fMRI mapping has the potential to add substantial value in the neurosurgical selection, consenting and planning of appropriately chosen patients. However, clinically meaningful fMRI mapping hinges on the precise identification of the surgical questions that fMRI is asked to inform, alongside careful characterization of patients to minimize fMRI studies in patients who are unlikely to benefit. It adds another layer of challenge for fMRI to provide ‘results’ when the question is poorly (or even not at all) defined beyond non-specific requests to “map ‘eloquent’ cortex”. Detailed knowledge and training are needed^181^ to evaluate fMRI results in the absence of “ground truth”, and meaningful clinical fMRI requires a highly interdisciplinary approach. When performed with due diligence and expertise, language fMRI mapping offers potential direct patient benefit. Examples of fMRI advantages include reducing the need for Wada testing,^5^ potentially enabling more aggressive or extensive surgical intervention when combined with tractography and ESM,^182–184^ and guiding optimal use of intraoperative stimulation^182,185^ and/or intracranial electrode placement,^186, 187^ especially, but not exclusively, in pediatric patients.

### 4.3. REQUIREMENTS FOR VALIDATION AND STANDARDIZATION

Reservations surrounding the clinical use of fMRI language mapping are derived largely from comparisons with the Wada procedure (for language lateralization) and ESM (for localization). Previous reviews have summarized the highly variable rates of sensitivity and specificity reported when fMRI results are compared to these clinical standard techniques.^21,42,188,189^ There are reasons to be cautious about interpreting such direct evaluations, not only because of factors such as brain shift, but especially because of differences in what each technique reflects and how results from each tool are derived.

ESM is considered a clinical standard tool for the preservation of brain function during surgery but, like all techniques, has limitations (further considered in https://doi.org/10.31219/osf.io/r7u8p). Not all patients are able to tolerate^190,191^ or complete ESM.^192^ There remains little standardization in both how ESM is executed^193^ and how stimulation-induced language errors are interpreted.^193,194^ Importantly, preservation of all ESM-positive language cortex does not absolutely prevent enduring language deficits following epilepsy^195^ or tumor surgery, especially in high-risk locations such as the inferior parietal cortex (16.7% long-term deficits in one study^96^). **Alternative methods, able to *pre*-operatively predict surgical risk and the likely safe(st) surgical approach, therefore retain an important role in neurosurgical planning**. Among such methods, fMRI is most used, likely because it offers the greatest amount of spatial detail and does so non-invasively. However, fMRI specificity is *per se* limited compared to ESM. More areas are typically activated in fMRI than are associated with language errors during ESM, resulting in only modest overall specificity (approximately 55%-71%) when grouping across populations and approaches.^189,196^

It is important to contextualize these results: by temporarily evoking or inhibiting behavioral responses, **Wada testing and ESM assess language function in fundamentally different ways from fMRI**. Often, pre-operative fMRI paradigms do not probe the same components of language, or do so to different degrees than Wada and intra-operative ESM. For example, silent word generation tasks optimized to control for non-linguistic motoric aspects of word generation will by design *not* predict sites that cause speech arrest when stimulated intra-operatively.^71^ Instead, fMRI offers distinct advantages to evaluate crossed dominance for different aspects of language that are difficult to evaluate through Wada testing, and to explore language processing at the systems-level, including contributions of contralateral brain structures that are inaccessible to ESM. Neither Wada nor ESM preclude (long-term) postoperative deficits (e.g.,^96,179,197,198^). Still, the expectation – wrongly – prevails that correspondence between these techniques must achieve 100% for fMRI to be useful. Like others before us, we advocate that **the clinical usefulness of fMRI should be evaluated in terms of its ability to anticipate and minimize (further) post-operative language decline, especially for patients unable to tolerate or complete awake surgery**.^190^

In this regard, preoperative language fMRI mapping predicts postoperative performance on tests of naming^5,199^ and verbal memory^5,199,200^ in patients following dominant temporal lobe surgery for epilepsy. The predictive value for post-surgical outcomes appears superior for language laterality as determined by fMRI than by Wada,^5,200,201^ although these studies are hampered by varying approaches for interpreting the Wada, typically ignoring mixed dominance possibilities. In small case series, post-operative language deficits arose in patients whose resection area spatially overlapped with pre-operative fMRI activations^202–204^ even when no overlap occurred with positive ESM sites.^198^ Diverging results are also reported.^205^ A recent study reported better 3-year survival rates in high grade (but not low grade) glioma and metastasis patients operated with fMRI than without fMRI, irrespective of the use of ESM.^206^ This result was ascribed to greater surgeon confidence in undertaking extensive resections when guided by fMRI, as highlighted in previous reports.^179^ Thus, studies examining the predictive value of fMRI for avoiding postoperative language decline generally support fMRI’s complementary role in surgical planning. Still, **no technique on its own is infallible** and **the best patient outcomes likely result from a combined approach**.^198^ Reliability of speech and language fMRI mapping depends on the nature of the pathology and is likely to differ between epileptic lesions, brain tumors and vascular malformations. In high-grade brain tumors and high-flow AVMs, pathological vessels *per se* may only contribute within different limits to the oxygen supply of the parenchyma, somewhat restricting the use of fMRI (and Wada testing). Consequently, more comprehensive longitudinal evaluations of the applications that fMRI is particularly valuable for, and where its prediction failed, remain needed.

### 4.4. VISION(S) FOR IMPROVING CLINICAL FMRI

International standardization of practice and reporting is needed to direct large-scale studies to objectively evaluate the long-term benefits of clinical language fMRI across neurosurgical indications. Emerging technological advances that offer higher resolution acquisitions, and the adoption of sophisticated preprocessing and analysis methods from the research realm, both hold promise for more precise localization of clinical fMRI results. Additional research is advocated to compare individual tasks head-to-head in terms of the strength and robustness of their associated activation patterns, and reliability in the context of patient performance difficulties. A particular limitation in validating language fMRI mapping is the sparsity of data in patients with atypical language organization. Due to the rarity of atypical language dominance, reports to date have largely consisted of case studies or very small case-series from which it is difficult to draw firm conclusions as to the accuracy and limitations of fMRI. Additionally, few studies have investigated how common medications, such as antiepileptic and antiedematous drugs (e.g.,^207–209^), may adversely affect BOLD fMRI. Finally, since all clinical tests carry some level of error, further consideration should be given to the level of evidence required to evaluate fMRI as a clinical standard for select indications. We propose this discussion should move towards detailed evaluation of pre- vs post-operative speech and language deficits, as determined through patient-reported and validated, standardized neuropsychological outcome measures across the wide range of patient populations routinely undergoing clinical fMRI.

## Figures and Tables

**Table 1.** Preferential roles and lesion consequences of proposed core language regions

Gyrus/Area	Subregion	Prevalent* language process	Predicted** consequence of damage
Inferior frontal (IFG)	Pars opercularis (pop)	**Phonological processing** & articulatory control	• Intraoperative stimulation^†^: **Anomia & phonological paraphasias** • Excision / infarct *‡*: Reduced (phonemic) fluency; ‘Broca’s **aphasia’**
	Pars triangularis (ptr)	**Semantic executive control,** esp. **under high demands**	•Intraoperative stimulation: **Anomia** • Excision / infarct: Disrupted speech and perhaps comprehension; (Semantic) errors during naming or reading
	Pars orbitalis (por)	**Controlled semantic retrieval**	• Intraoperatively: Most likely no behavioral change • Excision / infarct: Sentence comprehension; semantic errors in reading
	Ventral premotor cortex	**Speech output / articulation**	• Intraoperatively: **Dys/anarthria** • Excision: Speech production deficits
Middle frontal (MFG)	Mid-to-posterior, including inferior frontal junction	**Semantic processing**; **task** & **language switching**	• Intraoperatively^†^: semantic errors & paraphasias; anomia • Excision / infarct: transient deficits
	“Area 55b”; junction with precentral gyrus	**Articulation, fluency**; integration of phonological & semantic processing	• Intraoperatively: Speech arrest, semantic paraphasias, anomia • Excision / infarct: apraxia of speech
	“Exner’s”; junction of SFS and precentral gyrus	**Graphemic** **representations / control**	• Intraoperatively: **Agraphia** (writing arrest/errors) • Excision / infarct: **Agraphia**
Superior frontal (SFG)	pre-Supplementary Motor Area (SMA)	**Initiation and sequencing of spontaneous speech**	• Intraoperatively: Speech slowing / arrest, naming/fluency errors • Excision: Ataxic speech
Insula	Insula	**Complex articulatory control**	• Intraoperatively: Speech arrest/anarthria, anomia • Excision / infarct: (persistent) **aphasia**, reduced **fluency**
Inferior Parietal Lobe; TemporoParietal junction (IPL/TPJ)	General region	**Cognitive processes required for writing**	• Intraoperatively: **Writing errors/agraphia** • Excision / infarct: Pure or apraxic **agraphia**
	Supramarginal gyrus (SMG)	**Phonological processing** with sub-regions (e.g., for concept integration)	• Intraoperatively: **Anomia, phonological paraphasias / articulation** errors • Excision / infarct: “Broca’s aphasia”
	Angular gyrus	**Multimodal executive semantic processing & integration** with subdivisions	• Intraoperatively: **anomia**, sometimes **reading** errors • Excision / infarct: **anomic** aphasia, Agraphia / dystypia / **alexia**, Sentence /auditory **comprehension** deficits
Lateral temporal lobe	Posterior superior temporal gyrus (STG) / sulcus (STS) (lateral to planum temporale)	**Phonological processing** incl. auditory short-term memory and feedback monitoring	• Intraoperatively: Anomia, Phonological paraphasias / repetition errors, **Comprehension deficits**, Semantic paraphasias • Excision / ischemia: **Wernicke’s aphasia**, Repetition errors, Broca’s aphasia, Naming errors, Syntactic difficulties
	Middle STG/STS (lateral to Heschl’s gyrus)	**Acoustic processing** of attended / perceived meaningful word sounds	• Intraoperatively: Anomia, phonological errors, Semantic paraphasias, **Sentence** / word comprehension deficits • Excision / ischemia: **Comprehension** deficits, especially **sentence**-level
	Posterior middle temporal gyrus (MTG)	**Multimodal semantic processing & integration** (Lexical-semantic & Semantic-phonological)	• Intraoperatively: **Anomia**/dysnomia, **Semantic**, **phonological**, reading errors • Excision / ischemia: Comprehension, **naming** errors, **Semantic** / **phonological** paraphasias/ errors
	Middle MTG	**Word-level semantic processing**	• Intraoperatively: Semantic and phonemic paraphasias; **Auditory single word comprehension** • Excision / ischemia: **Auditory comprehension**, naming errors
	Posterior) lateral ITG	**Lexical access** for words; **integrating word sound & meaning**	• Intraoperatively: **Anomia** and **alexia** • Excision / ischemia: **Naming** errors and (long-term) **alexia** (if white matter involved)
	Temporal pole (ant. STG + MTG)	**Semantic combinations / judgements** for complex sentence structure	• Intraoperatively: **Naming** / sentence **comprehension** errors • Excision / ischemia: Word finding difficulties, esp. visual **naming,** Word and sentence **comprehension** deficits
Medial inferior temporal	Ventral occipitotemporal cortex (VOTC) (mid- post. fusiform)	**Orthographic processing** with subdivisions (visual categorization vs integration)	• Intraoperatively: Reading errors /disruption esp. meaning-based (morphogram) **reading** • Excision / ischemia: Impaired **reading**
	Basal temporal language area (BTLA) (ant. fusiform, between pole & VOTC)	Multimodal **semantic processing**; orthographic-semantic integration	• Intraoperatively: Various errors, most consistently in **naming** • Excision / ischemia: Persistent **naming** decline

This table is not intended to suggest one-to-one mapping of function, but rather reflects current evidence favoring relative graded representations of certain language-relevant computations across sub-regions of the brain.

* Some regions perform multiple computations, either specific to language or not.

** The true incidence / likelihood of subsequent deficits is very variable due to obvious variations in the nature of injuries/resections and individual patient factors; so ‘predicted consequences’ are intended to give an idea of the range of deficits that may arise, in the context of the language-related process most commonly associated with dissociable brain regions.

† The localization of stimulation-induced language errors, especially in the region of the posterior middle frontal gyrus, are difficult to ascribe to specific anatomical sub-regions due to the ambiguity of these functional boundaries and deformation of the surface anatomy by tumors.

‡ Similarly, infarcts generally cannot be precisely ascribed to specific cortical subregions due to their involvement of vascular territories that usually span more than one functional subregion. NOS = not otherwise specified. Supporting references, further granularity in basic language processes and less common stimulation-induced language errors / damage-related deficits are presented in the expanded version of this Table: https://doi.org/10.31219/osf.io/r7u8p

**Table 2.** A selection of language localization tasks according to surgical target

Target Area	Active condition	Main targeted process(es)	Control condition	Pros / Cons	Adaptations
**Pars Opercularis**	(Covert) verb generation*	Phonological processing	Silent rehearsal (e.g., numbers) or repetition	Easier than fluency tasks but no language response measure	• Auditory / visual • Rate of stimulus delivery (e.g. from 3s (hard) to 6s (easy)
	Sentence completion	All, including syntax	False fonts (rearranged letter parts)	Relatively easy, more lateralizing than word tasks, but no language response measure	• Auditory / visual • Rate & duration of sentence presentation
	(Covert) naming, e.g., object-sentence generation	Phonological & semantic processing	Silent counting (to control for motor planning) or rest	Simple, similar to intra-operative tests, but difficult for patients with anomia	• Remove items not consistently named • Substitute rhyming or repetition task if severe anomia
	(Covert) phonemic / semantic fluency	Phonological, Executive control	Silent rehearsal or repetition	Cognitively demanding, no language response measure	• Auditory / visual • Frequency of letter targets (e.g. 1 (hard) to 5-6 (easy) targets per block)
**Pars triangularis**	Sentence completion	Sentence level semantic processing, phonological processing	Visual scrambled letters / auditory reversed speech	No response measure, difficult for alexic patients or needs aural delivery system	• Auditory / visual • Rate of stimulus presentation (fewer sentences per block)
**Pars orbitalis**	Semantic description decision task	Complex associative semantic processing	Visual line drawings / auditory reversed speech	Button box response, but default version requires auditory system	• Auditory / visual • Adapt to performance level (e.g., children)
**ventral Premotor Cortex**	Overt articulation / covert word generation / naming	Articulation and phonemic	Rest	Direct comparator to intraoperative speech but increases head motion confounds	• Auditory / visual
**Mid-to-posterior MFG**	Sentence completion	Semantic and phonological processing	Visual scrambled letters / auditory reversed speech	No language response measure, difficult for alexic patients / need auditory system	• Auditory / visual • Rate of stimulus presentation (fewer sentences per block)
	If bilingual: language switching (naming) task	Cognitive control	Rest	No language response measure	• Remove items not consistently named • Rate of picture presentation (fewer/slower)
**Area 55b**	Articulation/fluency	Phonemic/articulation	Rest	No performance measure	• Choose task according to abilities
**Exner’s area**	Writing dictated words	Graphemic	Insufficient data, possibly drawing non-word shapes	Technically challenging to implement in MRI	• None identified
**Pre-SMA** ^‡^	Verb generation	Phonological processing	Resting fixation	No behavioral measure	• As for Pop
**Insula**	Fluency	Phonological, Executive control	Resting fixation	No behavioral measure	• As for Pop
	Two object naming/sentence generation	Articulatory control	Rest	No behavioral measure (or needs a noise-cancelling microphone)	• Visual / auditory • Adjust pace or rate of stimulus presentation
**Intra Parietal Sulcus**	Writing (or a motor planning task might suffice)	Graphemic/goal-direction action planning	As for Exner’s	As for Exner’s	• As for Exner’s
**SMG**	Auditory / Written sentence comprehension	Phonological processing and semantic integration	Perceptual control, e.g., reversed speech/false font	No behavioral measure	• Speed, complexity or length of written word presentation
	Auditory / Written sentence completion	Sentence level semantic processing & phonological integration	False font / reversed speech	Relatively easy but no language response measure	• Auditory / visual • Rate & duration of sentence presentation
**Angular gyrus**	Visual semantic relatedness decision	Semantic processing	Decision on non-linguistic items (e.g., tones/lines)	Behavioral response, stronger semantic response for visual than aural stimuli^499^	• Present aurally for deaf patients
	Written sentence completion	Semantic processing (integrating written words into sentence level meaning)	False font sentences	Relatively easy but no language response measure	• Auditory / visual • Rate & duration of sentence presentation
**posterior STG**	(auditory) Sentence completion	Phonological processing & integration (phonological-semantic- syntactic processing),	Perceptual control (e.g., reversed speech / false font/symbol strings) AND resting blocks^†^.	Generally easy, but no response measure	• Visual / auditory • Adapt stimulus rate / length to (e.g., reading) abilities • For severe comprehension difficulties, use object sentence generation or story listening (vs reversed speech)
**middle STG**	Auditory naming	Semantic & Phonemic / phonological	Acoustic control (e.g., tones or reversed speech)	Requires dedicated hardware, no response measure	• Adjust stimulus rate and pace • Use object or written naming in deaf patients
	Auditory sentence completion	Semantic & Phonemic / phonological	Acoustic control (e.g., reversed speech)	Relatively easy but no language response measure	• Use visual stimuli for deaf patients • Vary rate & duration of sentence presentation
**posterior MTG**	(written) Sentence completion	Semantic integration (sentence level)	Visual control (e.g., scrambled letters)	No language response measure, difficult for patients with alexia/dyslexia	• Auditory version for patients with reading difficulties • Adjust to ability (reading level / pace)
**middle MTG**	Sentence completion	Semantic processing (word level)	Perceptual control (auditory/visual)	No language response measure	• Auditory/visual • Adjust to ability (reading level / pace)
	Semantic relatedness decision or categorization	Semantic processing (word level)	Auditory: tone decision; visual: non-semantic decision (e.g., font)	Semantic categorization is most validated, but relatedness tasks more lateralizing	• Auditory or visual
**posterior ITG**	Written sentence completion	Lexical-semantic/orthographic processing	Visual control (e.g., scrambled sentences)	No language response measure, difficult for patients with alexia/dyslexia	• Adjust to ability (reading level / pace) • Use single words instead of sentences
	Word or sentence reading	Lexical-semantic/orthographic	Visual control (e.g., scrambled letters)	No language response measure, difficult for patients with alexia/dyslexia	• Adjust to ability (reading level / pace) • Use single words instead of sentences
**Temporal Pole**	Semantic relatedness decision or categorization task	Semantic	Perceptual control: tone (auditory) or non-linguistic visual decision (e.g. upper case/lower case font)	Auditory version needs hardware, sentence decisions more lateralizing than word decisions	• Visual / auditory • Adjust stimulus presentation (slower or fewer per block)
	Auditory description decision task	Semantic	Tone decision	Button box response, but default version requires auditory system	• Auditory / visual • Adapt content to comprehension level • Slow down stimulus pace
**VOTC**	Sentence reading	Reading	Rest and consonant, false font or symbol strings	Difficult for patients with visual field deficits/dyslexia/alexia, pseudo- or non-word letter strings can reduce relevant activation	• Vary complexity (short or longer) sentences, adjust to reading age / performance level
**BTLA**	Naming (read-response / picture)	Semantic processing & phonological retrieval	Scrambled images / false-font text scanning	Generally easy but challenging for patients with visual impairments	• Adjust stimuli to performance (slower rate / easier stimuli) • remove pictures not reliably named

A range of published fMRI language tasks that probe the predominant function(s) of anatomical regions of surgical concern. Tasks are not intended to be prescriptive, but offer examples of tasks that engage specific language processes most commonly affected by damage or disruption to a given brain region (see Table 1). Similarly, other control conditions are possible, depending on the clinical question.

* Covert generation is recommended to minimize head motion; if mapping of articulation-related sites (e.g., ventral premotor cortex) is needed, overt speech compared to resting fixation should be considered.

† Some control conditions engage or de-activate language-related areas, or heighten attention/cognitive processing; inclusion of additional rest blocks to examine (de)activations of both active and control conditions is strongly encouraged.

‡ Inclusion of a motor task may help to define boundaries between the supplementary motor area and pre-SMA.

## Data Availability

Not applicable.

## Box 1. Summary recommendations for Clinical Language fMRI mapping

### Patient selection & preparation (section 2.1)

- **Obtain medical history and prior neuroradiological imaging** to identify suitable candidates (Fig 1), and anticipate artifacts that may render fMRI futile or require sequence changes.
- **Obtain a minimum cognitive and neurological assessment (and detailed neuropsychological assessment whenever feasible)** before fMRI to ensure patients have (at least) minimum residual (including the visual /auditory) abilities necessary to perform the required tasks and to identify fMRI task adaptations needed to maximize successful performance.
- **Always practice tasks** before fMRI to ensure the patient’s full understanding and ability to perform the tasks as desired, including how and when to make any required responses.
- For **pediatric patients, a simulated session** is highly recommended.

### Clinical goals (Section 2.2)

- Within the multidisciplinary team, **clearly define the clinical question** to **select the appropriate combination of fMRI tasks** based on intended purpose and known structure-function relationships (Fig 4, Table 1).
- **In multilingual patients,** prioritize the patient’s main language but ideally involve each language needed in their everyday life (e.g., using a language **‘switching’ task**).

### Design considerations (Section 2.3)

- **Adjust task parameters** (modality, speed of presentation, difficulty level, etc.) to patients’ performance abilities (and/or developmental stage) as much as possible (Fig 6, online Box 4).
- **Select language and control conditions** to match the clinical question & anatomy (Table 2).
- **Consider the intended analysis**; optimal task designs vary accordingly.

### Acquisition (Section 3.2)

- **Use 3T whenever possible, a TR of 1-3 seconds and voxels no larger than 3.0mm isotropic**.
- **Inform the patient about** problems caused by **head movement; spend time on positioning and comfort**.
- **Monitor head motion** throughout the task; stop and repeat if necessary.
- **Use multiple short fMRI runs** rather than one long run to help minimize motion and fatigue.
- **Acquire fieldmap data** to correct for fMRI image distortions (Fig 7).
- **Monitor activation maps in real time**.
- **Collect real-time behavioral data** to confirm task performance.
- **Provide feedback** to the patient (e.g. over the intercom) after each task run
- **Communicate directly** and verbally with the surgeon or relevant team member regarding the conclusions that can be drawn from fMRI maps.

### Pre-processing and analysis (Section 3.3)

- **Quality control each step**: Distortion-correct images and examine the need for and effect of any retrospective motion correction steps.
- **Minimize spatial smoothing**, as functional zones may be <4mm apart or occupy one bank of a sulcus (Fig 8).
- **Inspect any automated image segmentations** to ensure the area of surgical interest is not accidentally removed from analysis.
- **Carefully inspect** (and, if needed, adjust) fMRI-to-structural **registrations.**
- **Consider potential delays in task performance, hemodynamic responses** or other sources of temporal variability of the fMRI signal (e.g., through supplementary and/or complementary temporal derivative modelling or independent component analyses)
- Consider statistical approaches to **minimize false negatives**.
- **Inspect results at a range of statistical thresholds** (including unthresholded).
- **If calculating LIs, focus on (various) language ROIs, inspect a range of thresholds, and** use LI calculation methods that combine a range of thresholds.

### Interpretation and reporting (Section 3.4)

- **Describe data quality, quality control steps and any interpretative challenges** (e.g., emphasize areas where signal loss / artifacts prevent detection of meaningful fMRI signals, especially around a lesion or the expected location of critical cortex).
- **Images are powerful and potentially misleading**. If the reporting specialist considers the results invalid, state so and omit images from the report. Otherwise, if intended for surgical planning, provide images showing proximity of lesions to critical areas.
- **Integrate complementary methods** (diffusion tractography, cerebrovascular reactivity mapping, perfusion, etc).

## Box 2. Considerations: tasks for lateralizing language functions

**Levels of evidence:** Numerous reviews and meta-analyses emphasize high overall correspondence in language laterality derived from task fMRI compared to Wada,^5,41,55,56^ but each emphasized extensive variability in fMRI practices. A recent Delphi survey highlighted that different tasks for lateralization may be suitable for different questions, and optimal fMRI tasks have not yet been established.^47^

Given limited data to quantify clinical levels of evidence for any single task, our approach to creating recommendations was informed by:

1. Dedicated review of neuroscientific studies engaging selected language processes, and how lateralized those processes are,
2. A published systematic review assessing the lateralization value of different fMRI tasks,^51^
3. Published data comparing laterality from fMRI tasks to the Wada test (but see section 4.3. for limitations with this approach)

**The following recommendations are not intended to be prescriptive**. Rather, our aim is to highlight a combination of tasks *most likely* to provide high sensitivity for language lateralization based on current understanding of language-brain relationships.

### Recommendation 1.

**Establishing language lateralization requires a combination of tasks**. Different language processes engage different networks of brain regions, and are lateralized to different extents (Fig 2). Our knowledge of these systems is continually evolving and optimal language task development is an area of active research. We advocate adopting established tasks but also further evaluating newly-emerging tasks. Laterality established through a combined analysis of several fMRI tasks has shown better correspondence to Wada laterality than laterality based on individual tasks analyzed on their own.^57^

### Recommendation 2:

Most everyday ‘language’ requires a combination of phonological, lexico-semantic and syntactic processing (in addition to non-linguistic operations, such as cognitive control and working memory). **Using at least one sentence-level task is therefore strongly recommended.** When considering approaches to language lateralization with fMRI, an historical tendency has been to categorize tasks into those probing the language ‘production network’ (aka ‘expressive’ tasks), ‘comprehension network’ (aka ‘receptive’ tasks) and ‘semantic network’. Since **most fMRI tasks engage several (if not all) of these networks to varying degrees**, there are practical and theoretical limits to categorizing tasks as one or the other ^58–60^. Furthermore, it is the **task contrast** to a specific control or baseline condition, that enables to study specific language processes (section 2.3.3.2). With this caveat in mind, some specific task approaches to consider include:

1. Language ‘production’*: Generating or producing language, aside from the articulatory system, involves a network of brain regions including the inferior frontal, supramarginal and superior temporal gyri. A systematic review identified **word generation tasks** as **strongly lateralizing**,^51^ especially when using an appropriate ‘active control’. Among studies comparing laterality derived from fMRI versus Wada, letter fluency (9 studies), category fluency (5 studies) and verb generation tasks (5 studies) have been the most evaluated.^41,55,56^ Letter fluency engages the frontal lobe more than category fluency, which instead engages the temporal lobe more (e.g.,^58^ noting also^59^). The choice of preferred task is therefore also influenced by the surgical target. Single word generation tasks produce activation that is more confined to the frontal lobes than sentence generation tasks (which additionally engage wider network regions including the temporal lobes^60^), and are therefore useful to consider when lateralization is required in the context of a surgical target in the frontal lobe. Patients can have restricted fluency for reasons other than language impairment^61^; verb generation tasks are more likely to sustain patient performance and, thereby, fMRI activation.
2. Language (particularly sentence) comprehension*: Understanding the meaning of language engages a widespread system including the lateral and ventral temporal lobe and angular gyrus in addition to the inferior frontal cortex ^65–67^. A systematic review identified **sentence comprehension tasks,** in particular, to be **strongly lateralizing**.^51^ Relatively fewer studies have compared laterality from sentence comprehension fMRI tasks with the Wada test. Three of 5 studies in meta-analyses used visually presented sentences with either reading or a semantic/syntactic decision (both compared to a perceptual control). Auditory versions (2 / 5 studies) generally focused on story listening compared to reversed speech, and tended to emphasize passive listening.^56^ Comprehension can also be assessed using a sentence completion task, in which participants generate a word to complete a sentence stem; or responsive naming, in which a phrase is heard or read (e.g., “long yellow fruit” and interpreted to generate a name: ‘banana’), thus combining language ‘comprehension’ and ‘production’. Such tasks are useful to include to establish lateralization of the wider temporo-parietal (and temporo-parieto-frontal) networks. Simple sentence processing such as reading or passive listening are less lateralizing than sentence comprehension tasks involving a semantic or syntactic decision^51^ and, therefore, not recommended for language lateralization, at least when used alone and in standard GLM-type analyses, unless limited patient abilities require it.
3. Tasks requiring a **semantic decision**, when paired with an active non-linguistic control task, engage additional regions of the anterior and inferior temporal cortex compared to word production and passive sentence listening tasks. Since the use-case for language lateralization is often temporal lobectomy, a word comprehension task involving a semantic decision is important to engage the anterior and basal temporal cortices. The most extensively studied approach contrasts a semantic decision condition, in which the participant decides if an aurally presented word fits a category (e.g., “found in the USA” and “used by people”), with a nonlinguistic tone perception condition.^50^ In an alternative approach, pairs of words are presented and patients decide if the words are related (e.g., synonyms). In the visual domain, cued naming with action generation (e.g., pen = ‘writing’) can elicit semantic decisions on single words.^51^ Evaluations against Wada have mostly involved single word decision tasks, presented either visually or aurally (5 studies^56^). In a systematic review of fMRI tasks,^51^ **semantic tasks involving a relatedness decision** (e.g., word pairs) **were more strongly lateralizing than single word decisions**. However, lateralization power of semantic tasks is strongly influenced by choices in the fMRI baseline condition, which should be both active and involve a perceptual decision to best isolate linguistic semantic processing (Fig 5).

#### Modifications for language lateralization in patients with specific impairments are detailed in the full document (for additional considerations in pediatric patients, please also see section 2.3.6). Summary considerations are:

- **Visual impairments:** Some patients are blind/have visual field deficits. Others have better auditory than visual attention, and vice versa. When technically feasible (Appendix C), tasks using the auditory modality are crucial for some patients, and beneficial for others.^52,53,62^
- **If sentence level processing is impaired**, word-level tasks^‡^ are one option. Verb generation or single word comprehension (semantic decision) use frequent external cues (e.g., one item every few seconds), which facilitates performance in patients with selective restrictions in executive functioning. Another approach, depending on the nature of the patient’s difficulties, is to adopt passive story listening / sentence reading, which, however, are less strongly lateralizing in general.^51^
- **In patients with a/dyslexia**, object naming can engage widely distributed lexico-semantic and phonological processing. However, lateralization in naming tasks depends on what ‘picture naming’ is contrasted against.^51^ It is essential to include a perceptual control (e.g., involving a visual detection/decision on complex un-nameable images) to isolate semantic and phonological components of naming tasks.
- **In patients with anomia:** Some studies^63,64^ have evaluated tasks using image pairs (e.g., an object and a separate written word). Further research is needed to evaluate if such “sentence generation” tasks are easier for patients with naming impairments to perform, but one recent study showed this approach^65^ may offer better lateralization than single object naming.

* Of emerging interest (although likely reserved for cognitively high performing patients) are grammar tasks. Tasks assessing syntax or morphology engage brain regions involved in hierarchical sentence-level operations (e.g., posterior middle temporal gyrus^66,67^), potentially more robustly than simpler lexico-semantic tasks.^45^

^‡^ Visual rhyming was recommended for lateralization by the ASFNR^7^ but supporting evidence varies.^51^ Nonetheless, rhyming is easier for patients to perform and could be considered in patients unable to perform other tasks, or in pediatric populations.

## Box 3. Considerations when selecting task control conditions

- **Active control conditions are generally recommended** over a ‘resting’ or passive baseline to localize activation specific to language (rather than activation reflecting low level sensory, motor, or attention processes). See https://doi.org/10.31219/osf.io/r7u8p when planning to alternatively use a conjunction analysis approach to isolate a particular function (such as phonological retrieval) through different tasks performed in various sensory domains (e.g., hearing, reading, seeing).^113^
- Active non-linguistic control conditions can increase sensitivity because many language areas, particularly those involved in semantic processing, are active during ‘resting’ states.
- Active controls that differ significantly from the task condition (e.g., tone discrimination vs word comprehension) can be useful to improve laterality indices by removing non-specific bilateral activation in sensory and attention networks and to detect language-specific activations.^50^
- **Some control conditions** (e.g., presenting reversed speech or pseudowords) **engage phonological components of the language network**^114^ **and potentially heighten attention**^115^; their use can reduce relevant signals related to phonological processing. In such designs, also including resting fixation blocks may be useful to explore the main effect of both active and control conditions separately, to aid interpretation of active > control block comparisons. This may require more powerful / flexible analysis models than allowed by certain commercial fMRI analysis packages.
- **Cognitively demanding** control conditions **make tasks more complex** for patients to understand or remember. These can therefore be impractical for some patients with moderate to severe comprehension, memory, attention or compliance deficits, and may need extensive pre-scan practice.

## Box 6. Select areas for further evaluation and research

### 1. Tasks to ensure a robust language map

- In-depth **comparisons of activation maps derived from different well tested tasks** and task designs that target similar language processes.
- **Evaluation of newly emerging tasks** for their merits in more comprehensively engaging wider language networks.
- Greater **clinical evidence** supporting the **specificity** of particular ‘activated’ neuronal populations for a given language process.
- Patient preferences and useability of different **task adaptations.**
- Development of **naming stimuli** that consider **different cultural backgrounds**, including **appropriate norms in pediatric populations.**

### 2. Evaluating the utility of fMRI on long-term post-surgical outcomes

- **Pooling Data from patients with and without fMRI.** E.g. including those ineligible or from sites without fMRI, with harmonized protocols and surgical approaches.
- **Publishing unexpected outcomes.** E.g. cases where preserved language fMRI activations led to deficits or where damaged activation sites did not cause impairments.
- **Longitudinal scanning and Diffusion tractography:** To provide more specificity on the degree of damage to the language networks.
- **Comprehensive assessments of long-term outcomes:** Early postsurgical outcomes alone are insufficient, as limiting tumour resection could lower survival in some patients.

### 3. For improvements in acquisition, analysis and interpretation of fMRI

- Objective evaluation of different approaches to visualize fMRI results (e.g. levels of statistical thresholding).
- **Additional development of alternative thresholding approaches** that offer variable control not only over false positive, but importantly also over potential false negative findings.
- Transparent **reporting** of clinical evidence testing the predictive value of specific fMRI task designs for **language outcomes**, especially in patients undergoing tailored lesionectomies.
- Specific value would be gained from **pooling of data**, especially across sites using fMRI alongside focal disruption techniques such as navigated TMS and / or ESM. Such endeavors are currently hindered by restrictions surrounding sharing of data collected in clinical settings, and by resource considerations (in relation to both direct costs, and that of staff needed to collect standardized (potentially longer or different) datasets in clinical settings).

## Footnotes

1 ^a^ Critical, often called “eloquent”, which is, strictly speaking a mereological fallacy according to Bennet & Hacker,^210^ and also used in a much broader sense in the neurosurgical community.^211^

2 While it is generally interhemispheric *dominance* for language (and verbal memory) that is sought to be established prior to esp. epilepsy surgery, traditionally by (super-)selective Wada testing, it is the asymmetrical *lateralization* of activations that can be established by fMRI. Although fMRI cannot discriminate indispensable from expendable activations per se (and at the individual patient level), lateralization has been shown to be a useful surrogate to predict postsurgical outcome in sufficiently large patient samples.
